# Vehicle modeling for the analysis of the response of detectors based on inductive loops

**DOI:** 10.1371/journal.pone.0218631

**Published:** 2019-09-13

**Authors:** Ferran Mocholí Belenguer, Antonio Martínez Millana, Antonio Mocholí Salcedo, Victor Milián Sánchez

**Affiliations:** 1 Traffic Control Systems Group, ITACA Institute, Universitat Politècnica de València, Valencia, Spain; 2 SABIEN Group, ITACA Institute, Universitat Politècnica de València, Valencia, Spain; 3 Department of Electronic Engineering, ITACA Institute, Universitat Politècnica de València, Valencia, Spain; 4 Chemical and Nuclear Engineering Department, Institute of Industrial, Radiological and Environmental Safety, Universitat Politècnica de València, Valencia, Spain; Tongii University, CHINA

## Abstract

Magnetic loops are one of the most popular and used traffic sensors because of their widely extended technology and simple mode of operation. Nevertheless, very simple models have been traditionally used to simulate the effect of the passage of vehicles on these loops. In general, vehicles have been considered simple rectangular metal plates located parallel to the ground plane at a certain height close to the vehicle chassis. However, with such a simple model, it is not possible to carry out a rigorous study to assess the performance of different models of vehicles with the aim of obtaining basic parameters such as the vehicle type, its speed or its direction in traffic. For this reason and because computer simulation and analysis have emerged as a priority in intelligent transportation systems (ITS), this paper aims to present a more complex vehicle model capable of characterizing vehicles as multiple metal plates of different sizes and heights, which will provide better results in virtual simulation environments. This type of modeling will be useful when reproducing the actual behavior of systems installed on roads based on inductive loops and will also facilitate vehicle classification and the extraction of basic traffic parameters.

## Introduction

The transformation of transport is a reality. New technologies applied to the automotive industry, big data and shared economy are changing the way people approach the world of transport. These advances, which are expected to contribute to an increase in the vehicle fleet together with the growth of the world population, will soon result in unsustainable traffic in the main cities, if no actions are taken in this regard [1]. Therefore, the need to have greater control over vehicles is increasing, which is why the extraction of information and the identification and classification of vehicles in real time emerges as a priority in our days within the intelligent systems of transportation (ITS).

All of the above implies continuous installation and improvement of the current road infrastructure, which is constantly redefining itself. Only a few years ago, the current infrastructure was limited to only physical components such as barriers, traffic lights and traffic regulators. However, the future road infrastructure will be forced to include components such as wireless networks, artificial intelligence and new sensor prototypes to adapt to the current technological changes. In addition, as roads cover a large proportion of the earth’s surface, especially within cities, the expected future is that the large number of emerging technologies can turn this element, now passive, into something much more productive.

This above consideration is the main reason why the simulation and characterization of vehicles over magnetic loops has currently become a field of much interest in ITS [2]. In the very near future, these capabilities should be able to identify the type and even the model of a vehicle depending on the detected magnetic profile, which goes beyond counting vehicles. Consequently, the number of applications will increase considerably. Simple but very effective examples would include the control of access to urban centers through bollards for pollution control or anti-terrorism purposes, anti-theft vehicle systems or obtaining much more reliable road parameters for statistical purposes.

However, although road infrastructure has changed significantly in recent years due to the continuous evolution of the technology, the truth is that magnetic loops continue to be the standard traffic sensor [3–8]. Currently, loop detectors still dominate traffic installations and are even part of the newest algorithms for traffic management in cities [9–11]. Moreover, these detectors have proven to be very cost effective and truly complete sensors since aside from their main application of vehicle classification, which includes buses, trucks, cars, motorcycles and even bicycles [12–17], magnetic loops are also used for vehicle speed measurements [18–24], wheel detection [25,26], bidirectional communication between vehicles and infrastructures [27] and vehicle re-identification [28].

**Fig 1:**
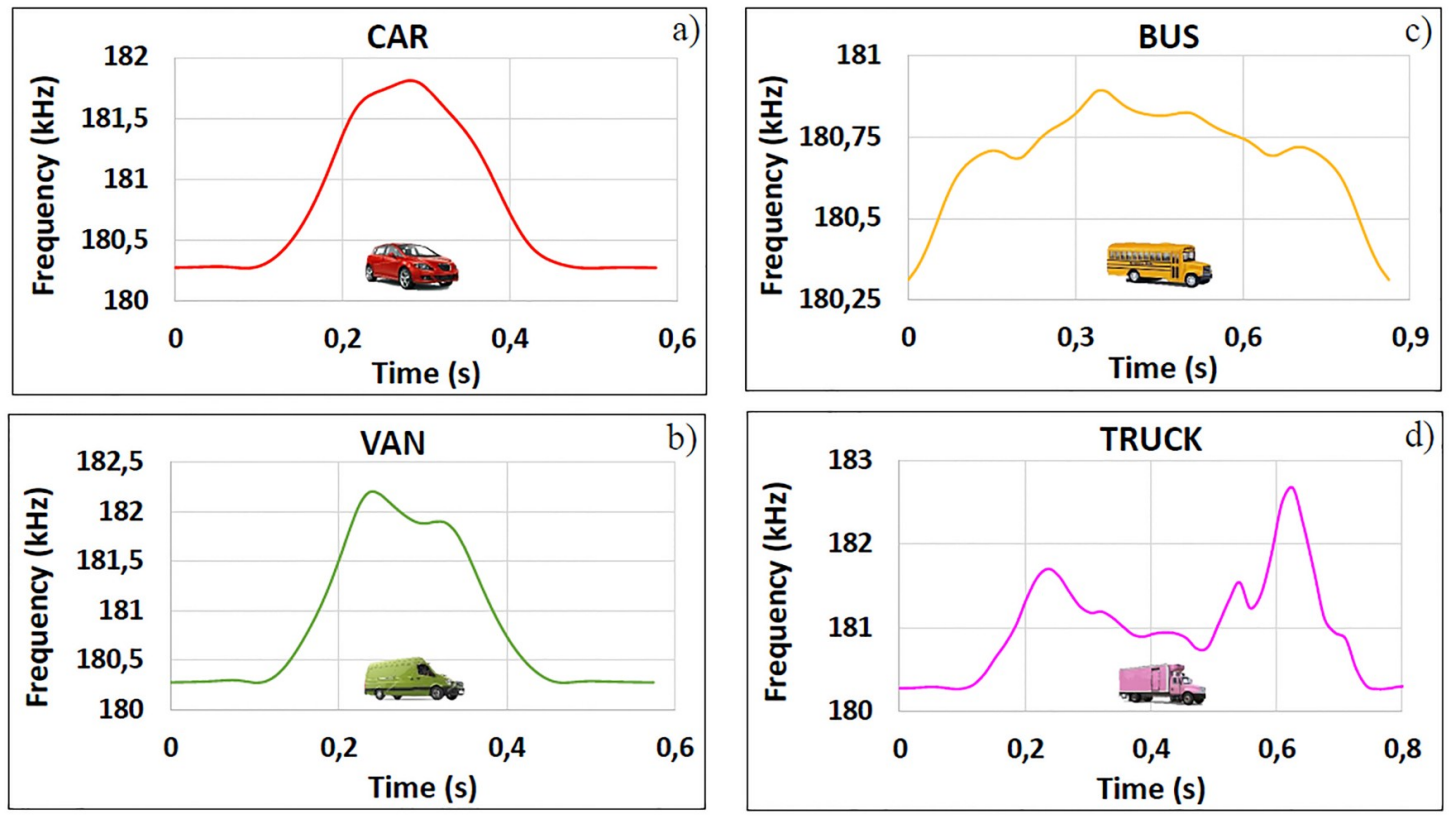
Real inductance signatures for (a) a car, (b) a van, (c) a bus and (d) a truck.

One of the most important aspects when simulating the passage of vehicles over magnetic loops is the analysis of inductance signatures, also called vehicle magnetic profiles. These signatures are the vehicle waveforms produced when vehicles pass over the loop detectors, and they are obtained by analyzing the changes in the frequency or inductance produced in the loop [29]. In this way, when a vehicle or any object constructed with a conductive material passes through its magnetic field, this decreases due to the currents induced in the vehicle, which also produce a decrease in the loop inductance. In addition, a very interesting peculiarity is that these magnetic profiles depend on parameters related to the particular vehicle, such as length, engine position or number of axes. Therefore, the profiles are different for each type of vehicle, as seen in Fig 1, and can be counted and classified in real time.

**Fig 2:**
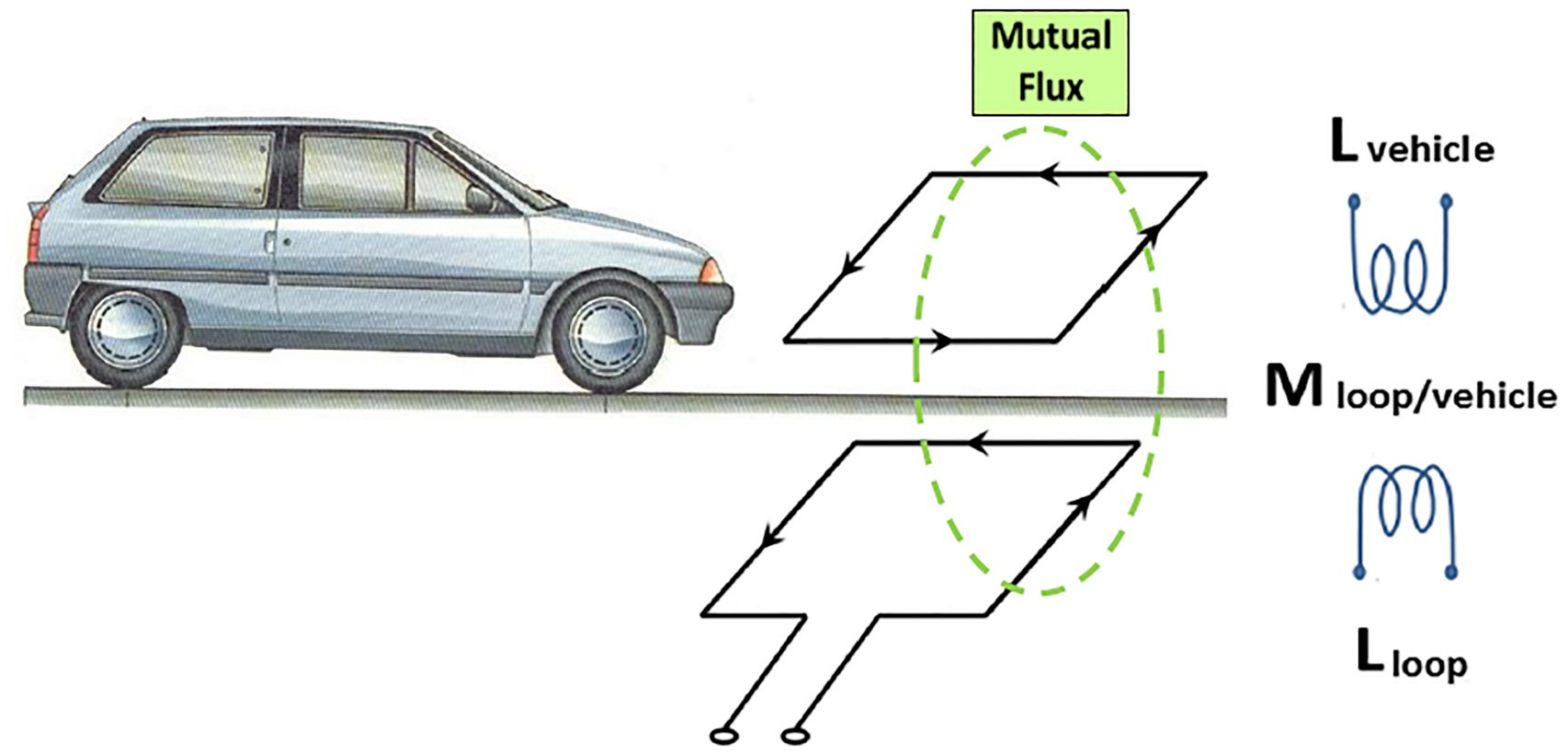
Model of a vehicle passing over the loop. The top represents the electronic model of the vehicle passing over the magnetic loop, which is represented at the bottom.

However, when trying to simulate the passage of vehicles over loops in computer programs, vehicles have traditionally been considered horizontal metal plates. For a long time, different authors [30] have supported this idea, and therefore, vehicles used to be modeled as rectangular metal plates whose width was equal to the width of the vehicle and whose length was equal to the length of the vehicle. Furthermore, these rectangular plates were simulated at a certain height from the ground, which corresponded to the average value of the height of the vehicle chassis. The electromagnetic behavior of this method is shown in Fig 2, where *L*_*loop*_ represents the inductance of the magnetic loop, *L*_*vehicle*_ represents the inductance of the metal plate that simulates the vehicle and *M*_*loop/vehicle*_ represents the mutual inductance between them.

But this simulation model used until today only proves useful for a first estimation. The model enables an increase in the oscillation frequency when some fictitious vehicle passes over an imaginary inductive loop, but it cannot offer much more. Thus, when developing an application in which the simulation of the real behavior of the loop-vehicle system is required, clearly, several limitations appear if this type of vehicle modeling is used.

An illustrative and clear example would be to simulate the passage of a rectangular metal plate over a conventional loop and observe that the result would be the same regardless of whether the plate moves in one direction or the opposite direction. Nevertheless, when real magnetic profiles are observed, it can be noted that in general, this is not true because of the asymmetries related to the vehicle structure, such as the position of the engine or the symmetry axes [31].

In vehicles with small dimensions, the result is usually a waveform that resembles a parabola, as shown in Fig 1a. In vehicles such as vans, the inductance signature is generally a waveform composed of two peaks, with the first being slightly larger, as shown in Fig 1b. In large vehicles such as buses or trucks, multiple peaks distributed along the magnetic profile are observed, as shown in Fig 1c and 1d, which are the result of the existence of distributions of different metal masses along the vehicle.

**Fig 3:**
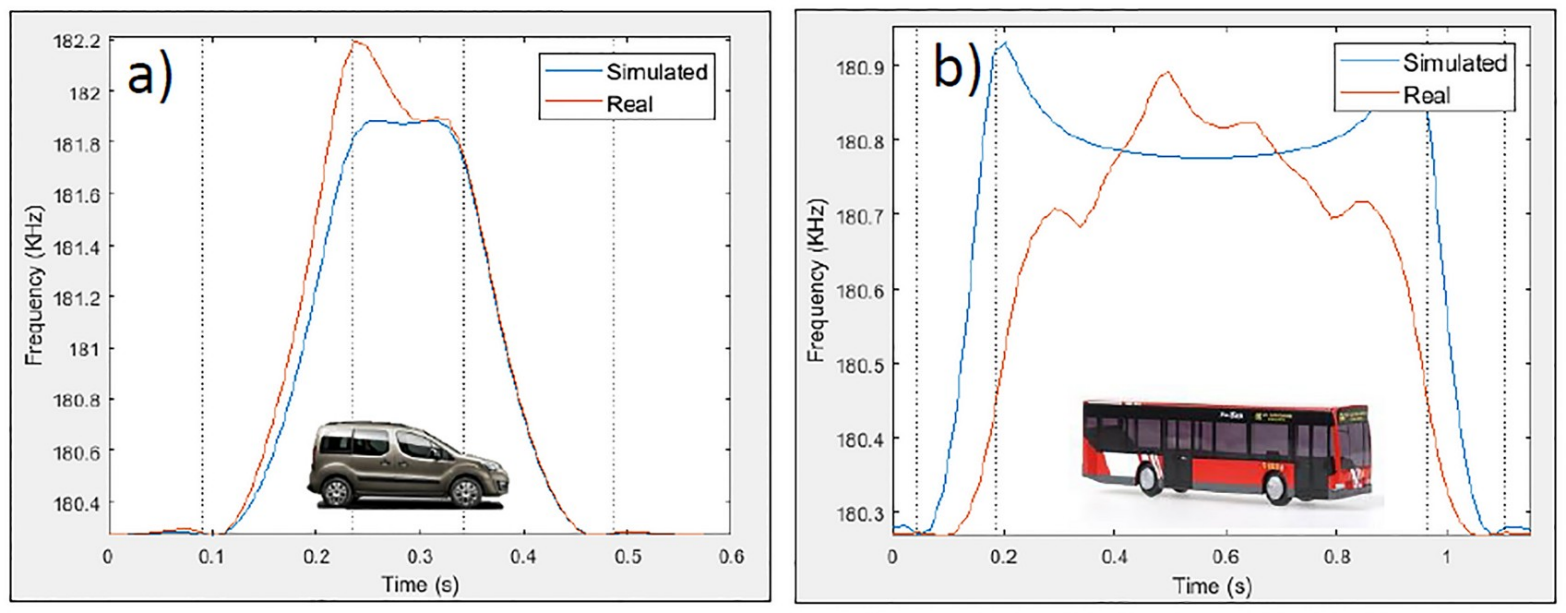
Differences between simulated and real magnetic profiles. (a) Simulated and real magnetic profile—Van. (b) Simulated and real magnetic profile—Bus.

Therefore, when trying to simulate the actual behavior of magnetic loops by modeling vehicles as simple metal plates, there is only one parameter that can be used to represent these asymmetries. This parameter is the height of the metal plate while it is moving over the loop. Then, this procedure could be somewhat appropriate when working with small vehicles because it would allow the maximum of the magnetic profile to be shifted by adjusting the linear path of the plate and the height, which varies linearly along their displacement. However, when working with large vehicles, in which there are multiple peaks in their magnetic profiles, the problem is more complex, as the linear paths cannot be used and the height does not follow a linear variation. Fig 3 shows the difference between the simulated inductance signatures considering the vehicle as a simple metal plate and the real measured inductance signatures of the same vehicle.

Thus, by simply observing the previous figures, there is a necessity to create new models for vehicle characterization that are more complex and realistic than the current models and can respond to challenging situations. As reflected in Fig 3, modeling the vehicles as simple metal plates provides merely an approximation. Hence, the importance and motivation of our paper and the proposed model are presented below.

## Model proposed

After comparing the different magnetic profiles, it has been shown that when vehicles are modeled as rectangular metal plates, the simulation is not entirely accurate. For this reason, we propose a new simulation technique based on sectioning the vehicles into multiple metal plates. Nevertheless, when we planned to carry out vehicle modeling that represents the real behavior and approximates the actual magnetic profile, a number of issues emerged that must be taken into consideration:

- The model must be easy to implement.
- Vehicles generally present symmetry along their axes.
- Vehicle magnetic characteristics along its entire length must be considered.
- These magnetic characteristics are associated with their geometry and structural configuration.

As simulations by known algorithms should be effortless, the first premise was to use simple regular geometries, such as circles or rectangles, because three-dimensional structures have such computational complexity that their use is discouraged. For this reason, flat geometries were chosen. However, we focused on rectangular shapes, as they have symmetry along their axis, and the form of a vehicle resembles a rectangle more than a circle.

**Fig 4:**
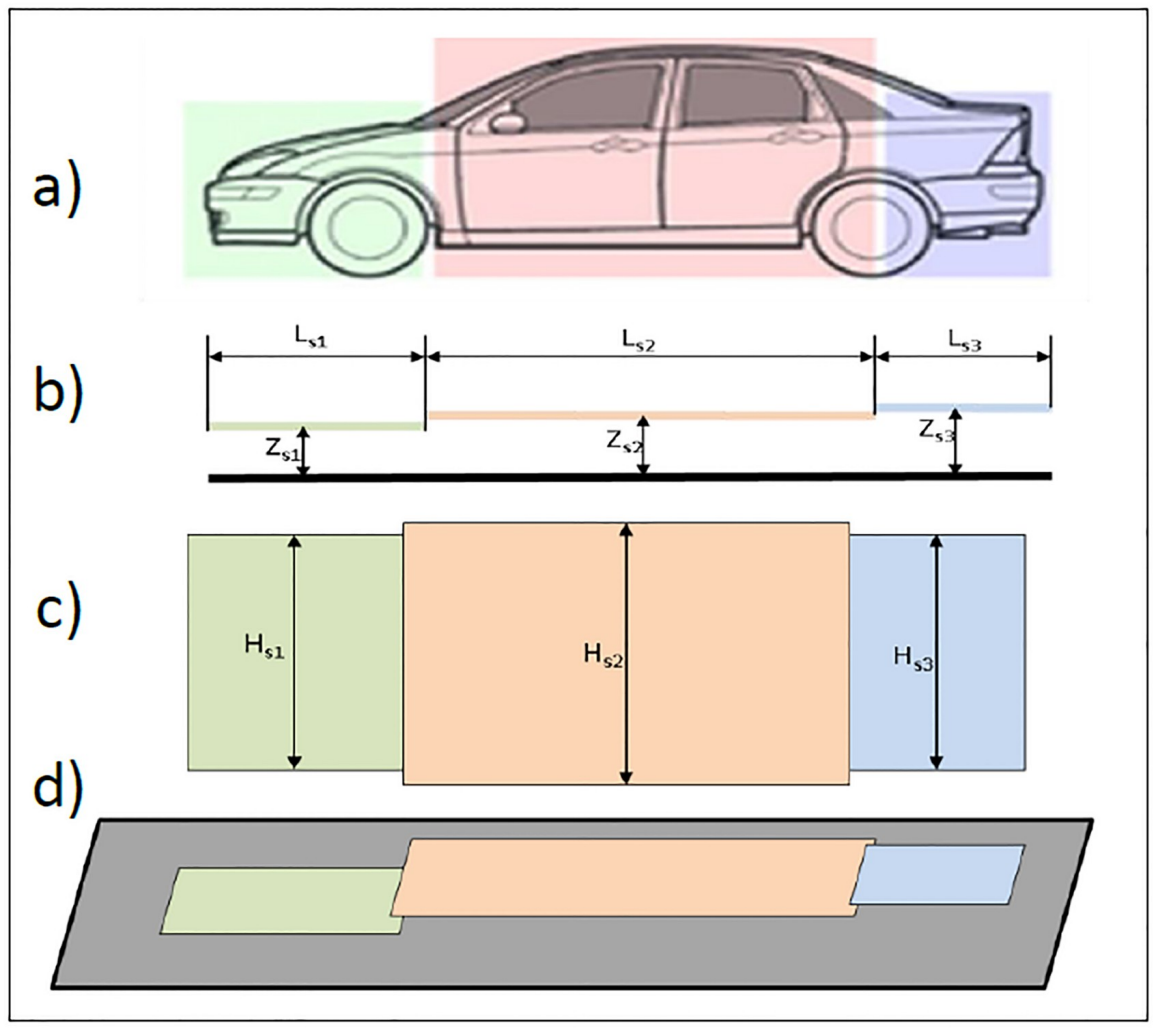
Vehicle modeled in three sections: (a) vehicle side view, (b) model side view, (c) model plan view and (d) model perspective view.

These issues led us to a vehicle model composed of multiple rectangles (multiple loops) in which each one represents a different section with a determined length, width and height. Hence, we will introduce how to calculate the inductance of the loop, the inductance equivalent to the modeling and the mutual inductance between them, which is exactly the operating principle shown in Fig 2. A visual example of this new modeling is shown in Fig 4, where a vehicle has been modeled in three different sections, namely, the engine area, the passenger area and the trunk area.

### Vehicle inductance

The inductance of a vehicle (*L*_*vehicle*_), i.e., its final value, is calculated by the sum of the individual values of the inductance of each of the isolated sections. In addition, with the purpose of giving even more flexibility to the system, we have considered the possibility that several sections of different lengths and widths located at the same height over the asphalt can be considered a single plane with a polygonal geometry and an arbitrary number of sides as long as it presents symmetry about the axis on which the vehicle is moving.

**Fig 5:**
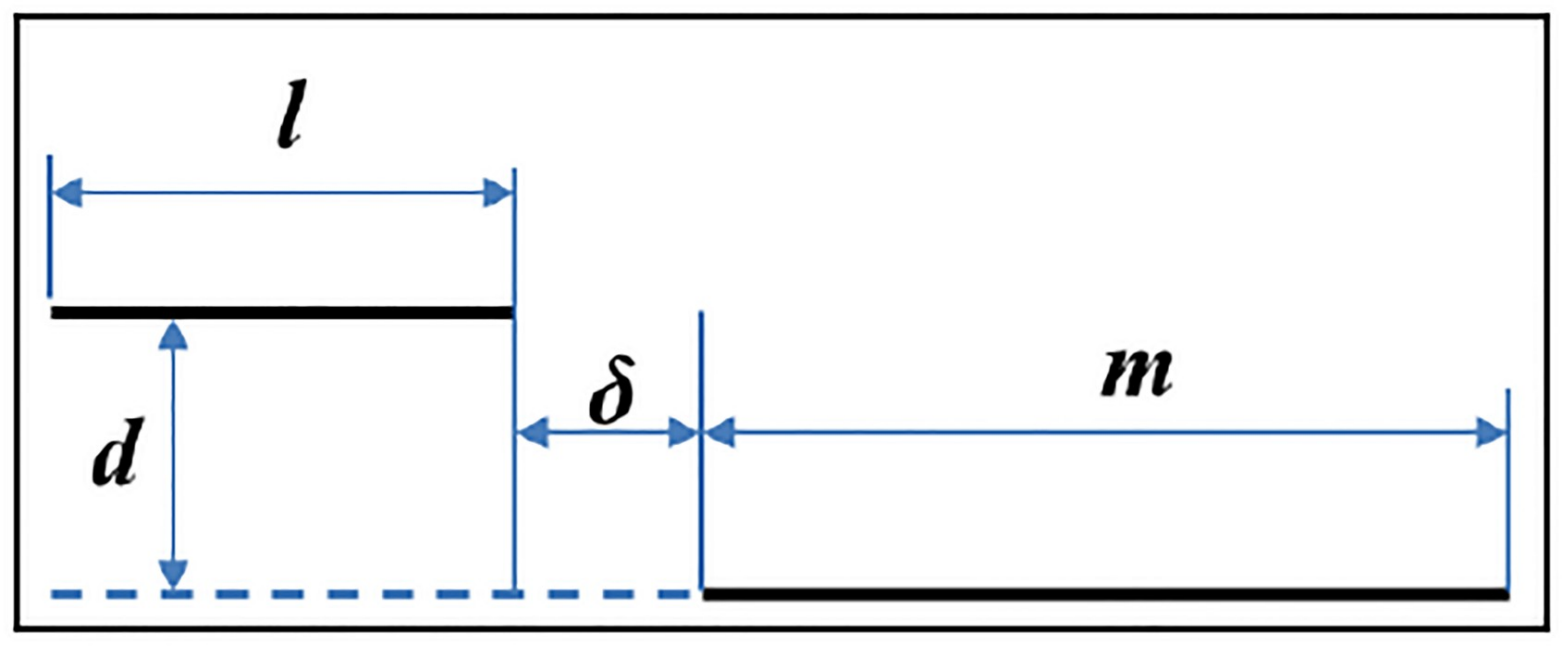
Scheme used to calculate the mutual inductance between two parallel conductors according to Grover’s equations.

This calculation will be based again on Grover’s equations [32,33], as in our previous papers [34–37], since they are capable of providing the value of the mutual inductance between two parallel rectilinear conductors with the geometry shown in Fig 5 with a very low computational cost.

In this way, according to Grover’s formulas, the mutual inductance *M*_*G*_(*l*, *m*, *d*, *δ*) between two parallel conductors of sizes *l* and *m* located between each other at a distance of *d* and displaced by a distance of δ, as shown in Fig 5, is given by:

$$\begin{array}{cc} M_{G}(l,m,d,\delta ) & =\frac{\mu_{0}}{4\pi }[\alpha \;{\text{sinh}}^{-1}\left(\frac{\alpha }{d}\right)-\beta \;{\text{sinh}}^{-1}\left(\frac{\beta }{d}\right)-\gamma \;{\text{sinh}}^{-1}(\frac{\gamma }{d})+\\ & +\delta \;{\text{sinh}}^{-1}(\frac{\delta }{d})\;-\;\sqrt{\alpha^{2}+d^{2}}+\;\sqrt{\beta^{2}+d^{2}}+\;\sqrt{\gamma^{2}+d^{2}}-\sqrt{\delta^{2}+d^{2}}] \end{array}$$

where the parameters *α*, *β* and *γ* are defined as:

α=l+m+δβ=l+δγ=m+δ

**Fig 6:**
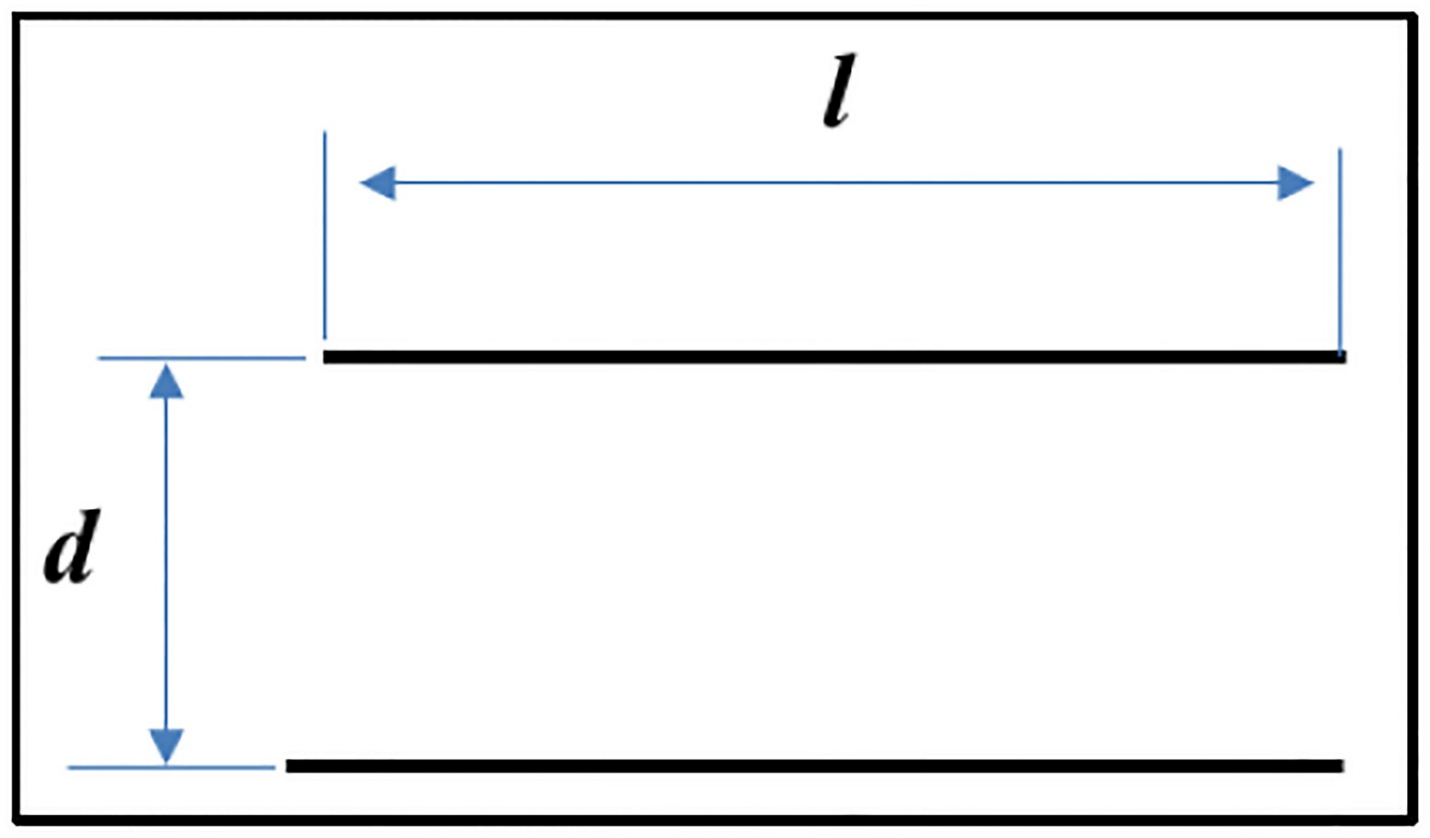
Two ideal parallel conductors.

In the event that the two conductors overlap partially or totally, the parameter δ will have negative values. Nevertheless, for the case of two parallel conductors, as shown in Fig 6, where *l* is the length of the filaments and *d* is the separation between them (both quantities expressed in meters to obtain the inductance expressed in Henrys), the expression can be simplified as:

$$M(l,d)=\pm \frac{\mu_{0}l}{2\pi }\;(ln[\frac{l}{d}+\sqrt{1+{\left(\frac{l}{d}\right)}^{2}}]-\sqrt{1+{\left(\frac{d}{l}\right)}^{2}}+\frac{d}{l})$$

This equation takes a positive sign when the current in both wires flows in the same direction and a negative sign when the currents flow in opposite directions.

If the different sections, in which the vehicle was divided, have different heights with respect to the plane of the roadway, then they are not be considered a loop with a polygonal geometry. In this case, the equivalent inductance of the vehicle would be obtained as the sum of each of the inductances of each section. This is:

$$L_{v}=\sum_{i=1}^{n_{S}}L_{0,j}\;$$

where *n*_*S*_ represents the number of sections in which the vehicle has been characterized and *L*_0,*j*_ represents the inductance of the section *j*.

**Fig 7:**
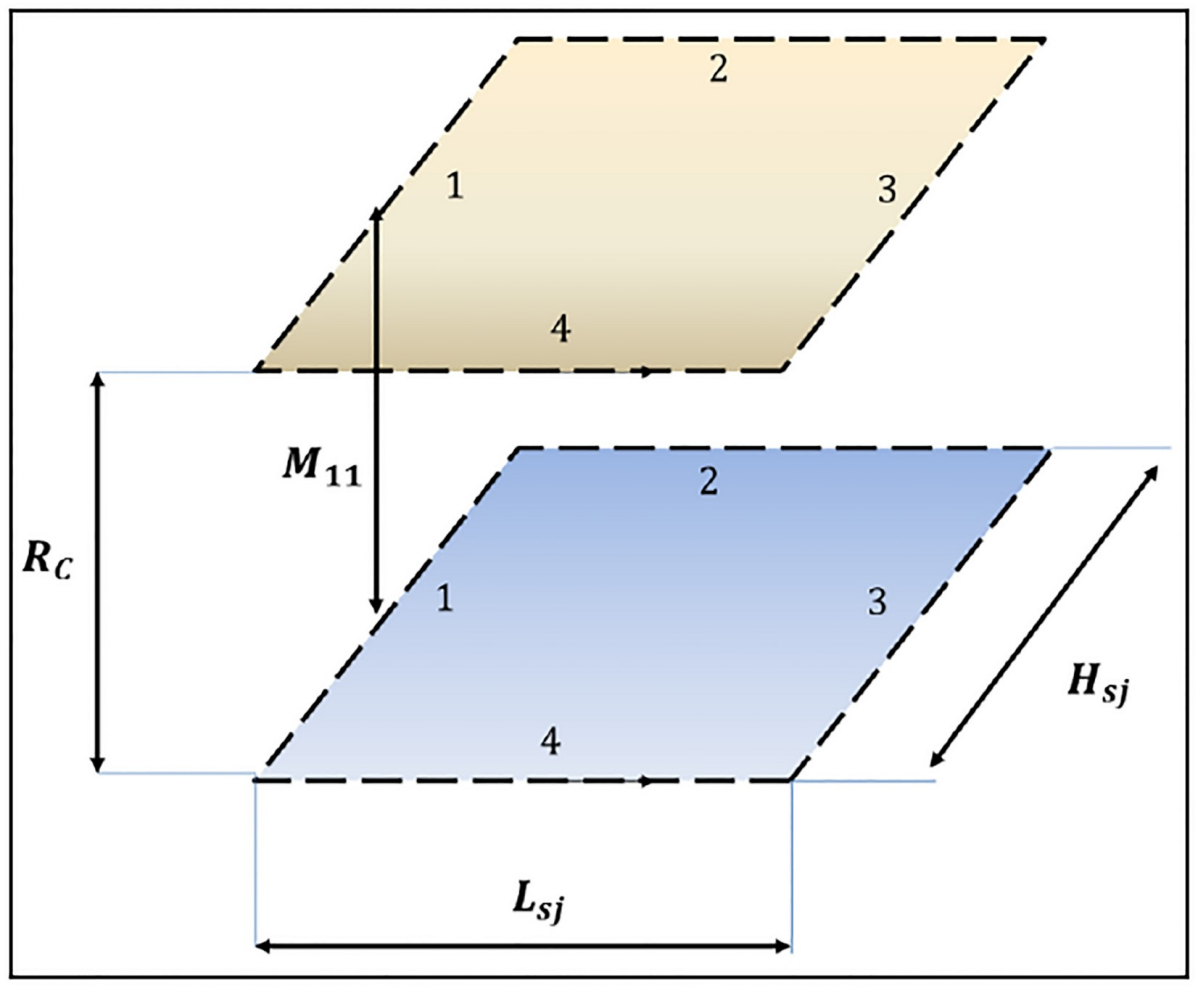
Geometry to calculate the mutual inductance between two parallel and coaxial rectangular loops with the same dimensions.

The inductance of each of the sections will be obtained as the sum of the internal self-inductance (*L*_0*i*,*j*_) and the external self-inductance (*L*_0*e*,*j*_) by using Mills and Grover’s equations particularized for single loops with only one turn as follows:

$${\;L}_{0,j}=L_{0i,j}+L_{0e,j}$$

where *L*_0*i*,*j*_ [32] is given by:

$${\;L}_{0i,j}=2(L_{sj}+H_{sj})L_{i}$$

where *L*_*i*_ the inductance per unit length, *L*_*sj*_ the distance of the filaments and *H*_*sj*_ is the separation between them, as shown in Fig 7.

**Fig 8:**
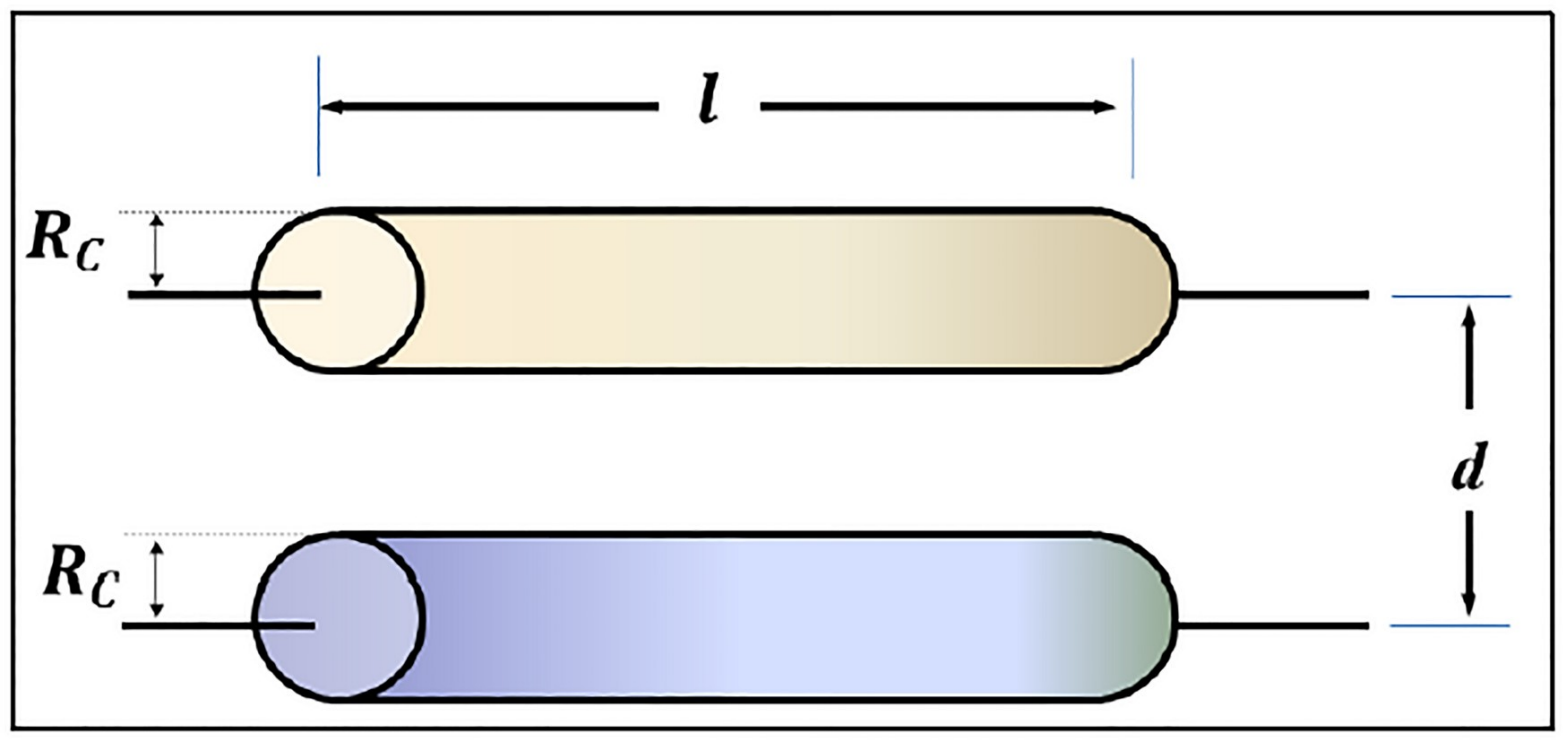
Features of two parallel conductors.

To calculate the external self-inductance of a conductor, a method in which the cable is replaced by two null-straight-section conductors separated by a distance *d* equal to the radius of the conductor *R*_*c*_ will be used. Therefore, the external inductance of a rectangular loop of one turn will be equal to the mutual inductance of two rectangular, parallel and identical coaxial loops separated by a distance equal to the radius of the conductor *R*_*c*_, as shown in Fig 8.

$$L_{P}=2(M(l,R_{C})-M(l,d))$$

Thus, the mutual inductance of two parallel rectangular loops, as shown in Fig 7, could be obtained from the mutual inductance values between parallel conductors. In this way, the mutual inductance between them (*L*_*sj*_ and *H*_*sj*_ of the representative loop of the section *j*), spaced at a distance *R*_*C*_ (this value is considered the plate thickness of the vehicle), could be expressed as:

$$L_{0e,j}=-2[M_{13}(H_{sj},\sqrt{{R_{C}}^{2}+{L_{sj}}^{2}})-M_{11}(H_{sj},R_{C})+M_{24}(L_{sj},\sqrt{{R_{C}}^{2}+{H_{sj}}^{2}})-M_{22}(L_{sj},R_{C})]$$

The terms *M*_*mn*_ represent the mutual inductance between the *m* segment of the low loop and the *n* segment of the top loop. The doubling of the equation is due to the mutual inductances, as they are all symmetric (*M*_*mn*_ = *M*_*nm*_).

Up to this point, it has been assumed that each of the sections was located at a different heights from the pavement. Consequently, each section was considered a single rectangular loop. However, with the aim of making this new vehicle model even more realistic and flexible, provided that they are of different sizes but are located at the same height, using polygonal sections of multiple parallel sides can be possible.

**Fig 9:**
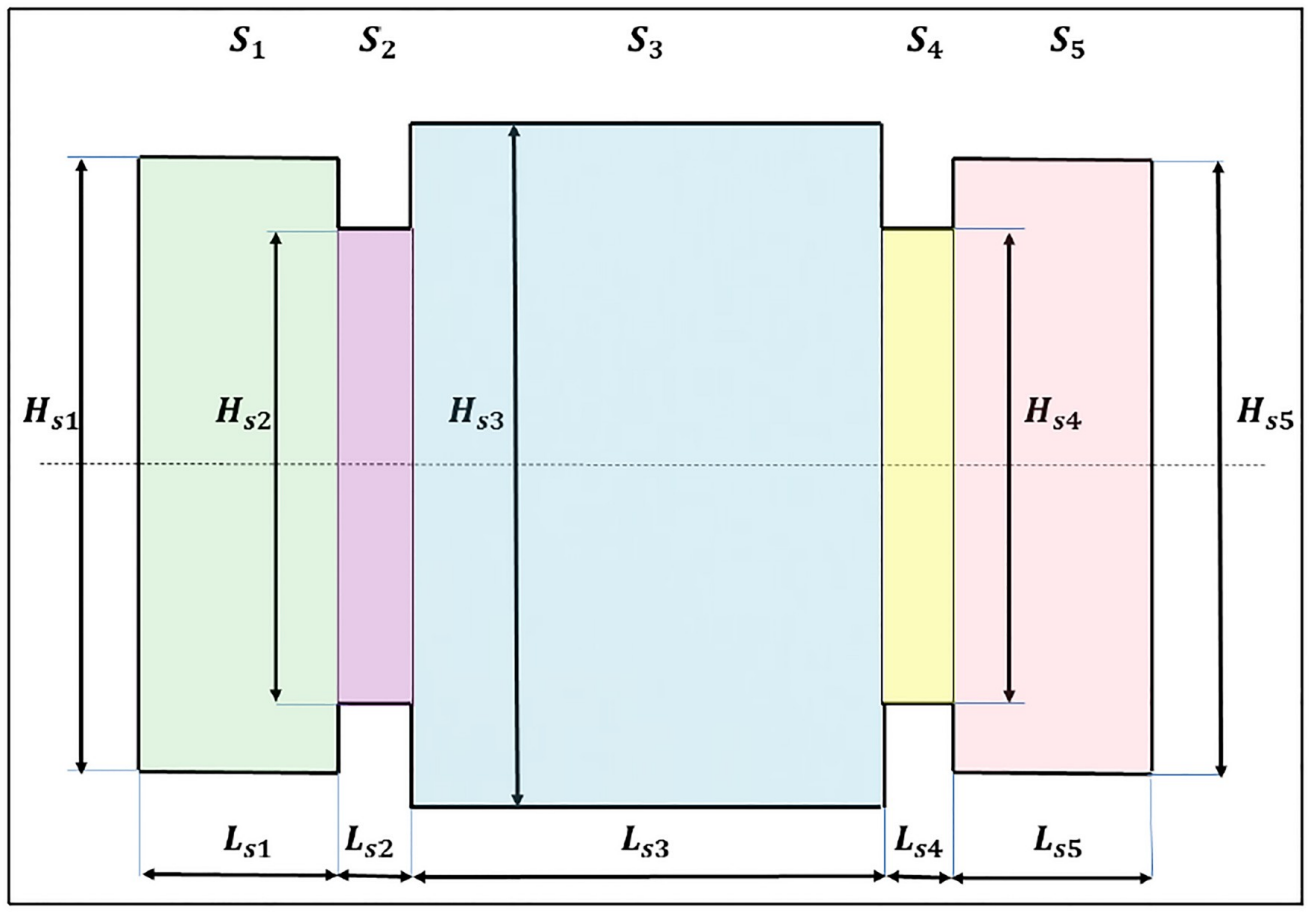
Vehicle modeled as multiple sections of the parallel sides of different dimensions but with the same height.

To analyze this type of modeling, such as the one shown in Fig 9, we will make use of Grover’s formula again, which we already know allows us to obtain the mutual inductance between parallel segments of different lengths and relative positions. Therefore, to obtain the total equivalent inductance *L*_0*T*_, the following equation must be applied:

$$L_{0T}=L_{0iT}+L_{0eT}$$

In this particular case, there are five sections, and it is assumed that they are located at the same height. Nevertheless, to perform the respective calculations, we will again divide the tasks into two parts: internal inductance and external inductance.

On the one hand, the calculation of the internal inductance will depend on the number of sections *n*_*S*_ and will be given by:

$$L_{0iT}=L_{i}\;(2*\sum_{m=1}^{n_{S}}L_{sm}+H_{s1}+H_{sn_{S}}+\sum_{n=2}^{n_{S}}Abs(H_{sn}-H_{s(n-1)})\;)$$

On the other hand, to obtain the external inductance, we will assume again that each conductor with a diameter of *R*_*C*_ is equivalent to two conductors of null thickness separated by a distance *R*_*C*_. Thus, the external inductance of the whole will be the mutual inductance between two parallel polygons spaced at a distance *R*_*C*_. To calculate this external inductance, we will begin calculating the mutual inductance between all horizontal conductors (*L*_0*eH*_), which can be expressed as:

$$\begin{array}{cc} L_{0eH} & =2\cdot (\sum_{m=1}^{n_{S}}\sum_{n=1}^{n_{S}}M_{G}(L_{sm},L_{sn},\sqrt{{\left(\frac{Abs(H_{sm}-H_{sn})}{2}\right)}^{2}+R_{C}^{2}},\sum_{p=min(m,n)}^{\text{max}\left(m,n\right)-1}L_{sp})\\ & -\sum_{m=1}^{n_{S}}\sum_{n=1}^{n_{S}}M_{G}(L_{sm},L_{sn},\sqrt{{\left(\frac{\left(H_{sm}+H_{sn}\right)}{2}\right)}^{2}+R_{C}^{2}},\sum_{p=min(m,n)}^{max(m,n)-1}L_{sp})) \end{array}$$

The next step will be the calculation of the mutual inductance between all vertical conductors (*L*_0*eV*_), whose value is given by the sum of several terms. First, we will calculate the inductance of the extreme sides of the polygon (*H*_*s*1_ and $H_{sn_{S}}$) as:

$$\begin{array}{cc} L_{0eVe} & =M_{G}(H_{s1},H_{s1},R_{C},-H_{s1})+M_{G}(H_{sn_{S}},H_{sn_{S}},R_{C},-H_{sn_{S}})\\ & -2M_{G}(H_{s1},H_{sn_{S}},\sqrt{{\left(\sum_{m=1}^{n_{S}}L_{sm}\right)}^{2}+R_{C}^{2}},-\frac{H_{s1}+H_{sn_{S}}}{2}) \end{array}$$

The following terms that must be taken into account will be those corresponding to the inductance between the extreme side 1 and each of the intermediate sections. This inductance will be expressed as *L*_0*eV*1_:

L0eV1=4∑m=2nS±MG(Hs1,Abs(Hsm-Hs(m-1))2,(∑n=1m-1Lsn)2+RC2,min(Hsm,Hs(m-1))-Hs12)

The plus and minus signs will depend on the relationship between the direction of an imaginary current that flows through the polyhedron and passes through the segment *H*_*s*1_ and the segment of length $\frac{Abs(H_{sm}-H_{s(m-1)})}{2}$, located between the sections *m* y *m* − 1. In this way, the plus sign will be taken when the directions of the currents in both segments are the same, which occurs when *H*_*sm*_ > *H*_*s*(*m*−1)_. Alternatively, the minus sign will be chosen when the directions of the currents in both segments are opposite, which occurs when *H*_*sm*_ < *H*_*s*(*m*−1)_.

Similarly, the terms that represent the inductance between the extreme side *n*_*S*_ and each of the intermediate sections will be expressed as:

L0eVnS=4∑m=2nS±MG(HsnS,Abs(Hsm-Hs(m-1))2,(∑n=mnSLsn)2+RC2,min(Hsm,Hs(m-1))-HsnS2)

In this second case, the plus and minus signs will also depend on the relationship between the directions of another imaginary current that flows through the polyhedron and passes through the segment $H_{sn_{S}}$ and the segment of length $\frac{Abs(H_{sm}-H_{s(m-1)})}{2}$, located between sections *m* y *m* − 1. In this case, the plus sign will be taken when the directions of the currents in both segments are the same, which occurs when *H*_*sm*_ < *H*_*s*(*m*−1)_, and the minus sign will be chosen when the directions of the currents in both segments are opposite, which occurs when *H*_*sm*_ > *H*_*s*(*m*−1)_.

Finally, we will have to add the terms that represent the mutual inductance between each pair of segments perpendicular to the axis (*L*_0*eVi*_), and as in the previous cases, Grover’s formula will be applied again, resulting in the following expression:

L0eVi=4·∑m=2nS-1∑n=m+1nS±(MG(Abs(Hsm-Hs(m-1))2,Abs(Hsn-Hs(n-1))2,(∑k=mn-1Lsn)2+RC2,min(Hsn,Hs(n-1))-max(Hsm,Hs(m-1))2)-MG(Abs(Hsm-Hs(m-1))2,Abs(Hsn-Hs(n-1))2,(∑k=mn-1Lsn)2+RC2,min(Hsn,Hs(n-1))+min(Hsm,Hs(m-1))2))

The plus and minus signs will also depend on the relative direction of the currents between the segments *m* and *n*. In this case, the positive sign will be used when *H*_*sn*_ < *H*_*s*(*n*−1)_ and *H*_*sm*_ < *H*_*s*(*m*−1)_ or when *H*_*sn*_ > *H*_*s*(*n*−1)_ and *H*_*sm*_ > *H*_*s*(*m*−1)_, while the negative sign will be taken in the other cases.

Therefore, the external vertical inductance will be given by the sum of all the previous terms:

$$L_{0eV}=L_{0eVe}+L_{0eV1}+L_{0eVn_{S}}+L_{0eVi}$$

Finally, the total external inductance will be given by the expression:

$$L_{0eT}=L_{0eH}+L_{0eV}$$

### Loop/Vehicle mutual inductance

To calculate the mutual inductance between the loop buried in the pavement and the vehicle modeled as multiple loops *M*_*loop/vehicle*_, it is considered that every single loop that represents the vehicle is independent of each other, without any mutual inductance between them.

**Fig 10:**
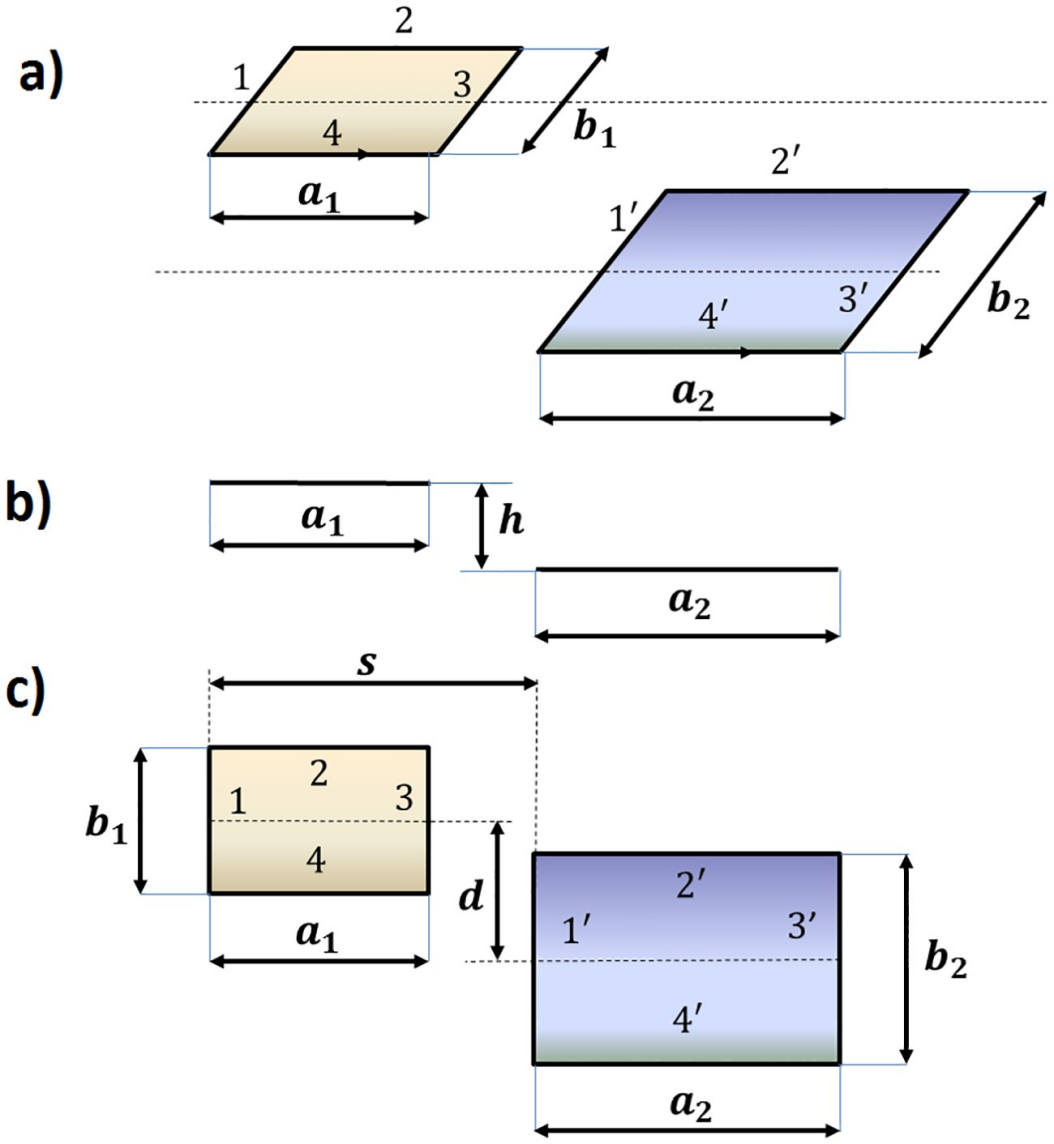
Two parallel and rectangular loops displaced longitudinally and laterally: (a) perspective view, (b) side view and (c) plan view.

In this method, loops are considered a superposition of *n*_*V*_ loops of one turn with a certain separation between them of *S*_*V*_. Then, with the aim of calculating the mutual inductance between the loop located on the road and the loops of the different sections of the vehicle, we should first introduce how to calculate the mutual inductance between two parallel and rectangular loops of different dimensions that are displaced both longitudinally and laterally, similar to those ones that are represented in Fig 10.

To perform this calculation, the mutual inductance between the different segments must be calculated and include the following:

1. Mutual inductance between segments 1 and 1’, whose value will be:
  $${M_{11^{'}}=M}_{G}(b_{1},b_{2},\sqrt{s^{2}+h^{2}},d-\frac{b_{1}+b_{2}}{2})$$
2. Mutual inductance between segments 3 and 3’, whose value will be:
  $${M_{33^{'}}=M}_{G}(b_{1},b_{2},\sqrt{{\left(s+a_{2}-a_{1}\right)}^{2}+h^{2}},d-\frac{b_{1}+b_{2}}{2}$$
3. Mutual inductance between segments 1 and 3’, whose value will be:
  $${M_{13'}=-M}_{G}(b_{1},b_{2},\sqrt{{\left(s+a_{2}\right)}^{2}+h^{2}},d-\frac{b_{1}+b_{2}}{2})$$
4. Mutual inductance between segments 3 and 1’, whose value will be:
  $${M_{31'}=-M}_{G}(b_{1},b_{2},\sqrt{{\left(a_{1}-s\right)}^{2}+h^{2}},d-\frac{b_{1}+b_{2}}{2})$$
5. Mutual inductance between segments 2 and 2’, whose value will be:
  $${M_{22'}=M}_{G}(a_{1},a_{2},\sqrt{{\left(d+\frac{b_{1}-b_{2}}{2}\right)}^{2}+h^{2}},s-a_{1})$$
6. Mutual inductance between segments 4 and 4’, whose value will be:
  $${M_{44'}=M}_{G}(a_{1},a_{2},\sqrt{{\left(d-\frac{b_{1}-b_{2}}{2}\right)}^{2}+h^{2}},s-a_{1})$$
7. Mutual inductance between segments 2 and 4’, whose value will be:
  $${M_{24'}=-M}_{G}(a_{1},a_{2},\sqrt{{\left(d+\frac{b_{1}+b_{2}}{2}\right)}^{2}+h^{2}},s-a_{1})$$
8. Mutual inductance between segments 4 and 2’, whose value will be:
  $${M_{42'}=-M}_{G}(a_{1},a_{2},\sqrt{{\left(d-\frac{b_{1}+b_{2}}{2}\right)}^{2}+h^{2}},s-a_{1})$$

Therefore, the global mutual inductance between both loops will be the sum of all of the inductance values between the segments:

$$M_{Ged}(a_{1},b_{1},a_{2},b_{2},s,h,d)=M_{11^{'}}+M_{33^{'}}+M_{13^{'}}+M_{31^{'}}+M_{22^{'}}+M_{44^{'}}+M_{24^{'}}+M_{42^{'}}$$

From these equations, clearly, the mutual inductance between the loop located on the road and each of the loops that simulate the vehicle can be calculated arbitrarily as the sum of all these inductances. However, if the case of a loop installed on the road of dimensions *a* × *b* centered at the origin of coordinates with a number of turns *n*_*V*_ separated by a distance *S*_*V*_ and a vehicle modeling with a number of rectangular sections *n*_*S*_ with a length of *L*_*V*_(*i*) and a width of *H*_*V*_(*i*) is considered, then the result of the mutual induction would be given by:

$$IM=\sum_{j=1}^{n_{V}}\sum_{i=1}^{n_{S}}M_{Ged}(a,b,L_{V}(i),H_{V}(i),s(i),Z(i)-(j-1)\cdot S_{V},d(i))$$

where:

- *s*(*i*) is the distance between the segment of the loop located on the road perpendicular to the axis of the displacement and the equivalent segment of section *i*.
- *Z*(*i*) is the height of section *i* of the vehicle modeling relative to the lower part of the loop buried in the pavement.
- *d*(*i*) is the lateral displacement between the axes of the loop located on the road and section *i* of the vehicle model.

**Fig 11:**
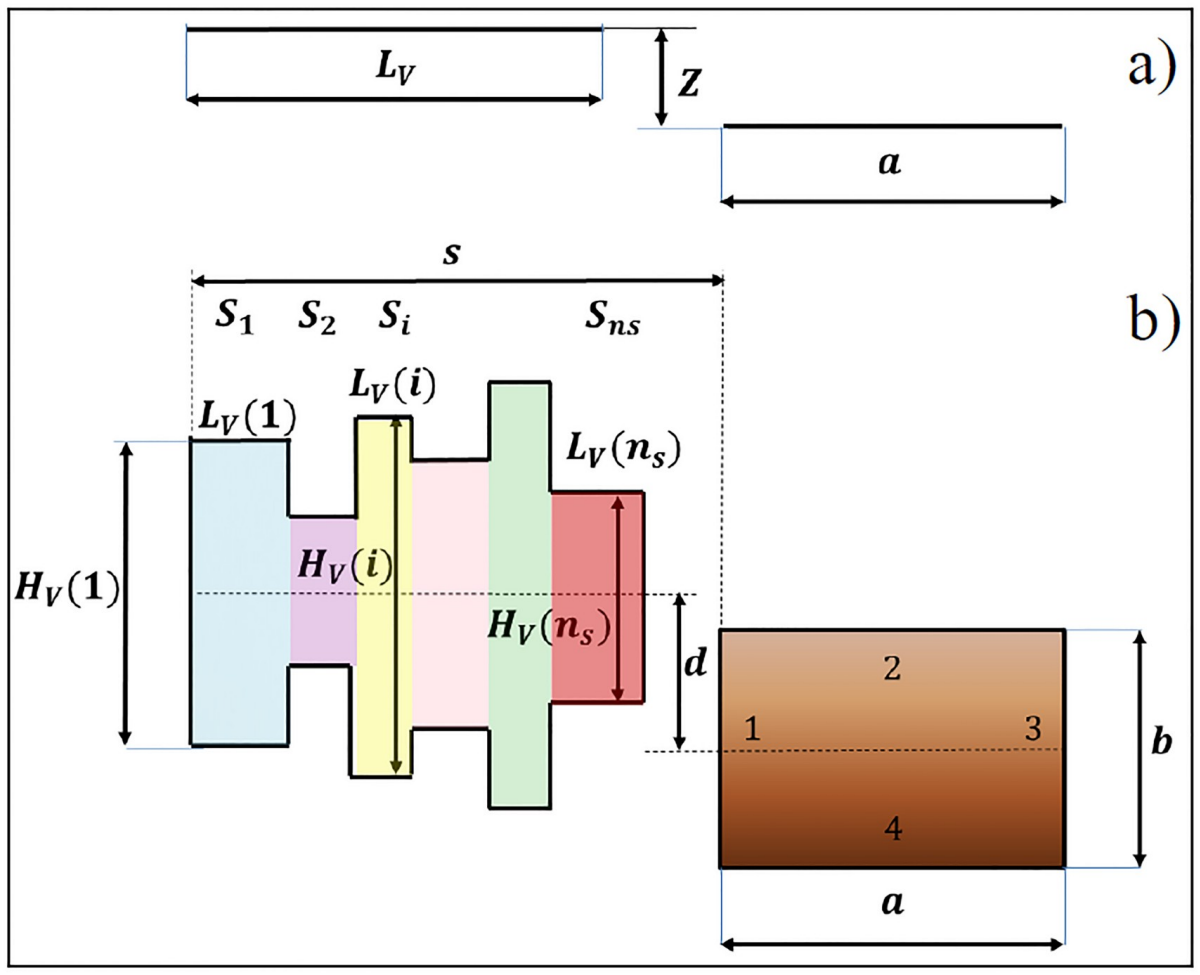
Representative diagram of the vehicle system for vehicles with polygonal forms and an arbitrary number of sections: (a) lateral view and (b) plan view.

Nevertheless, similar to how it was done for the calculation of the vehicle inductance, the case of contemplating polygonal surfaces will be analyzed again. In this situation, the figures would look like those shown in Fig 11.

We will begin with the mutual induction between the segments of each of the turns of the loop buried on the road perpendicular to the axis and each of the ends of the surface of the polygonal loop, which is:

$$M_{11}=\sum_{i=1}^{n_{V}}M_{G}(b,H_{V}(1),\sqrt{s^{2}+{\left(Z-(i-1)\cdot S_{V}\right)}^{2}},d-\frac{b+H_{V}(1)}{2})$$

$$M_{1n_{S}}=\sum_{i=1}^{n_{V}}M_{G}(b,H_{V}(n_{S}),\sqrt{{\left(s-L_{V}\right)}^{2}+{\left(Z-(i-1)\cdot S_{V}\right)}^{2}},d-\frac{b+H_{V}(n_{S})}{2})$$

$$M_{31}=\sum_{i=1}^{n_{V}}\;M_{G}(b,H_{V}(1),\sqrt{{\left(s+a\right)}^{2}+{\left(Z-(i-1)\cdot S_{V}\right)}^{2}},d-\frac{b+H_{V}(1)}{2})$$

$$M_{3n_{S}}=\sum_{i=1}^{n_{V}}M_{G}(b,H_{V}(n_{S}),\sqrt{{\left({\left(s-L_{V}\right)}^{2}+a\right)}^{2}+{\left(Z-(i-1)\cdot S_{V}\right)}^{2}},d-\frac{b+H_{V}(n_{S})}{2})$$

where:

- *b* is the width of the loop located on the road.
- *a* is the length of the loop located on the road.
- *d* is the lateral displacement between the loop located on the road and the vehicle loop.
- *H*_*V*_(1) is the width of the first section (1) of the polygonal surface of the vehicle loop.
- *H*_*V*_(*n*_*S*_) is the width of the last section (*n*_*S*_) of the polygonal surface of the vehicle loop.
- *Z* is the height at which the surface of the vehicle on the road is.
- *s* is the distance between the first vehicle loop segment and the first road loop segment.
- *L*_*V*_ is the total length of the vehicle ($L_{V}=\sum_{j=1}^{n_{S}}L_{V}(j)$).

Then, the mutual inductance between the segments of the loop perpendicular to the axis and each of the intermediate segments of the surface of the polygonal will be obtained as:

$$\begin{array}{cc} M_{inv} & =\sum_{j=1}^{n_{V}}\sum_{i=1}^{n_{S}-1}\pm (M_{G}(b,Abs(H_{V}(i+1)-H_{V}(i)),\sqrt{{\left(s-\sum_{j=1}^{i}L_{V}(j)\right)}^{2}+{\left(Z-(j-1)\cdot S_{V}\right)}^{2}},d+\frac{min(H_{V}(i+1),H_{V}(i))-b}{2})\\ & +M_{G}(b,Abs(H_{V}(i+1)-H_{V}(i)),\sqrt{{\left(s-\sum_{j=1}^{i}L_{V}(j)\right)}^{2}+{\left(Z-(j-1)\cdot S_{V}\right)}^{2}},d-\frac{max(H_{V}(i+1),H_{V}(i))+b}{2}))\\ & \mp (M_{G}(b,Abs(H_{V}(i+1)-H_{V}(i)),\sqrt{{\left(s+a-\sum_{j=1}^{i}L_{V}(j)\right)}^{2}+{\left(Z-(j-1)\cdot S_{V}\right)}^{2}},d+\frac{min(H_{V}(i+1),H_{V}(i))-b}{2})\\ & +M_{G}(b,Abs(H_{V}(i+1)-H_{V}(i)),\sqrt{{\left(s+a-\sum_{j=1}^{i}L_{V}(j)\right)}^{2}+{\left(Z-(j-1)\cdot S_{V}\right)}^{2}},d-\frac{max(H_{V}(i+1),H_{V}(i))+b}{2})) \end{array}$$

where *s* = *s*(1), i.e., the distance between the first segment of the vehicle loop and the first one of the loop located on the road.

To continue with the calculation, the mutual inductance between the segments of the loop parallel to the axis and each of the parallel segments to the axis of the surface loop will be calculated. This result is given by:

$$\begin{array}{cc} M_{inh} & =\sum_{j=1}^{n_{V}}\sum_{i=1}^{n_{S}}(M_{G}\;(a,L_{V}(i),\sqrt{{\left(d+\frac{H_{V}(i)-b}{2}\right)}^{2}+{\left(Z-(j-1)\cdot S_{V}\right)}^{2}},s-\sum_{j=1}^{i}L_{V}(j))\\ & -M_{G}\;(a,L_{V}(i),\sqrt{{\left(d-\frac{H_{V}(i)+b}{2}\right)}^{2}+{\left(Z-(j-1)\cdot S_{V}\right)}^{2}},s-\sum_{j=1}^{i}L_{V}(j))\\ & -M_{G}\;(a,L_{V}(i),\sqrt{{\left(d+\frac{H_{V}(i)+b}{2}\right)}^{2}+{\left(Z-(j-1)\cdot S_{V}\right)}^{2}},s-\sum_{j=1}^{i}L_{V}(j))\\ & +\;M_{G}\;(a,L_{V}(i),\sqrt{{\left(d+\frac{b-H_{V}(i)}{2}\right)}^{2}+{\left(Z-(j-1)\cdot S_{V}\right)}^{2}},s-\sum_{j=1}^{i}L_{V}(j))) \end{array}$$

Therefore, the global mutual inductance (*M*_*MG*_) between the loop located on the road and the polygonal surface that represents the vehicle will be the sum of the terms previously calculated:

$$M_{MG}=M_{11}+M_{1n_{S}}+M_{31}+M_{3n_{S}}+M_{inv}+M_{inh}$$

## Results

**Fig 12:**
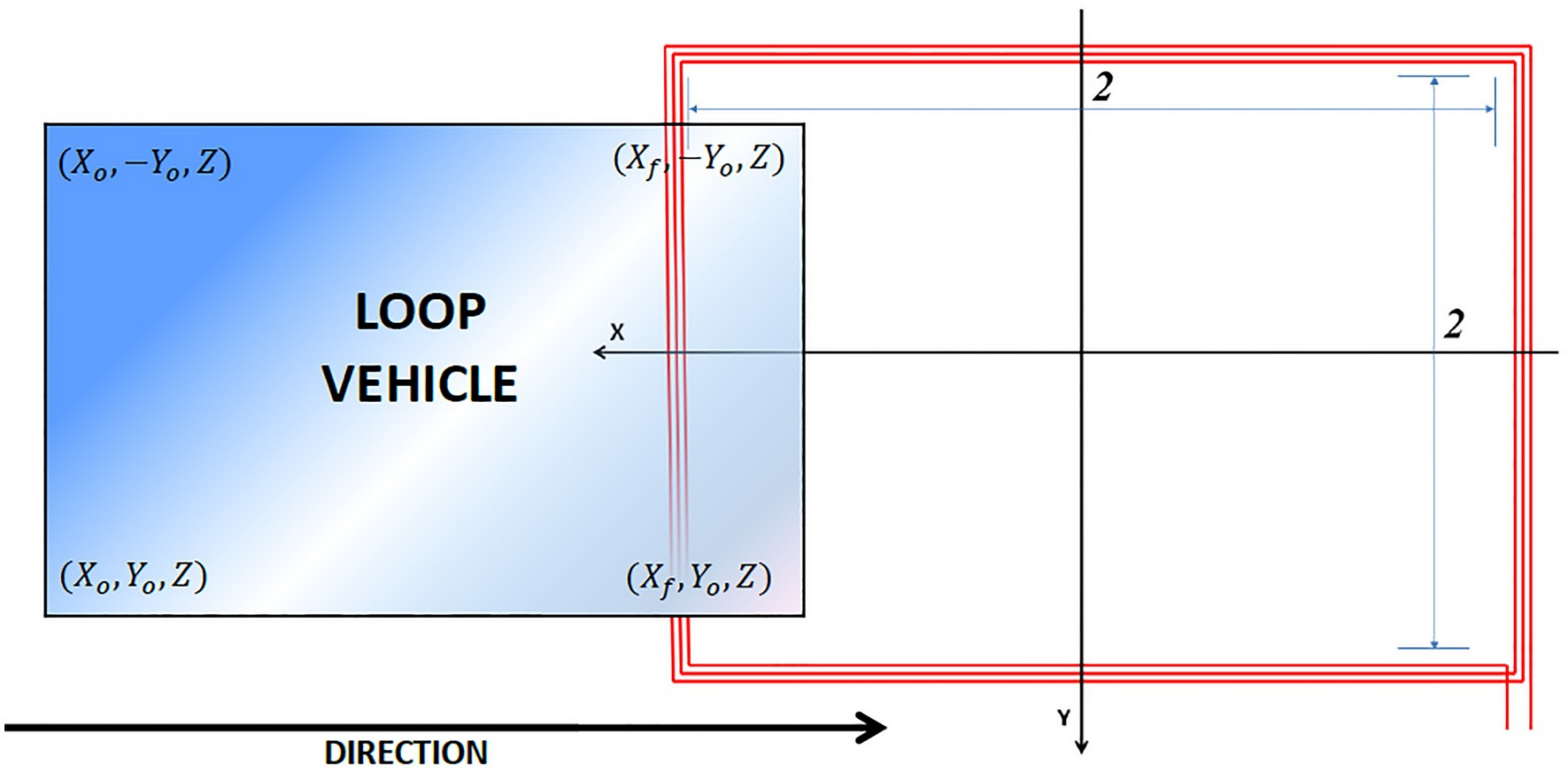
Equivalent vehicle model passing over the loop at a specific moment in time.

With the aim of proving the goodness of the presented vehicle modeling, it was necessary to develop a simulation program in both VisualBasic and MATLAB that is capable of presenting and comparing the real magnetic profiles generated by the passage of vehicles over standard magnetic loops with those simulated by using this new modeling. This program, designed by our research team (Group of Traffic Control System, ITACA Institute, Universitat Politècnica de València) and presented in [35,37], performs all the processes of the calculation and graphically presents the results and stores them in a file for both single and double loops [35,37]. However, a series of parameters must be introduced prior to the simulations. These parameters are as follows:

- The geometrical characteristics of the loop: dimensions according to the *X* and *Y*-axes.
- The type of copper conductor used, its radius and the current that will flow through it.
- The spacing between turns.
- The number of points used to calculate the self-induction of the loop according to the *X* and *Y*-axes for the numerical integration. If these points are not introduced, then the system assigns the values that have proven to be optimal by default.
- The number of turns of the loop. For single loops *N*_2_ = 0.
- The characteristics of the vehicle, which refers to its dimensions according to the three axes (length, width and height of the chassis over the asphalt). These characteristics allow the simulation of vehicles as rectangular single plates or considers them as several sections.
- The trajectory traversed by the center of the vehicle in the three directions of the space (*X*_*o*_, *X*_*f*_, *Y*_*o*_, *Z*), which can be observed in Fig 12.
- The speed at which the vehicle passes over the loop.
- The electrical characteristics of the components that constitute the oscillating circuit where the loop is incorporated. This oscillator circuit, which is involved in the value of the resonance frequency, is described in [36].

**Fig 13:**
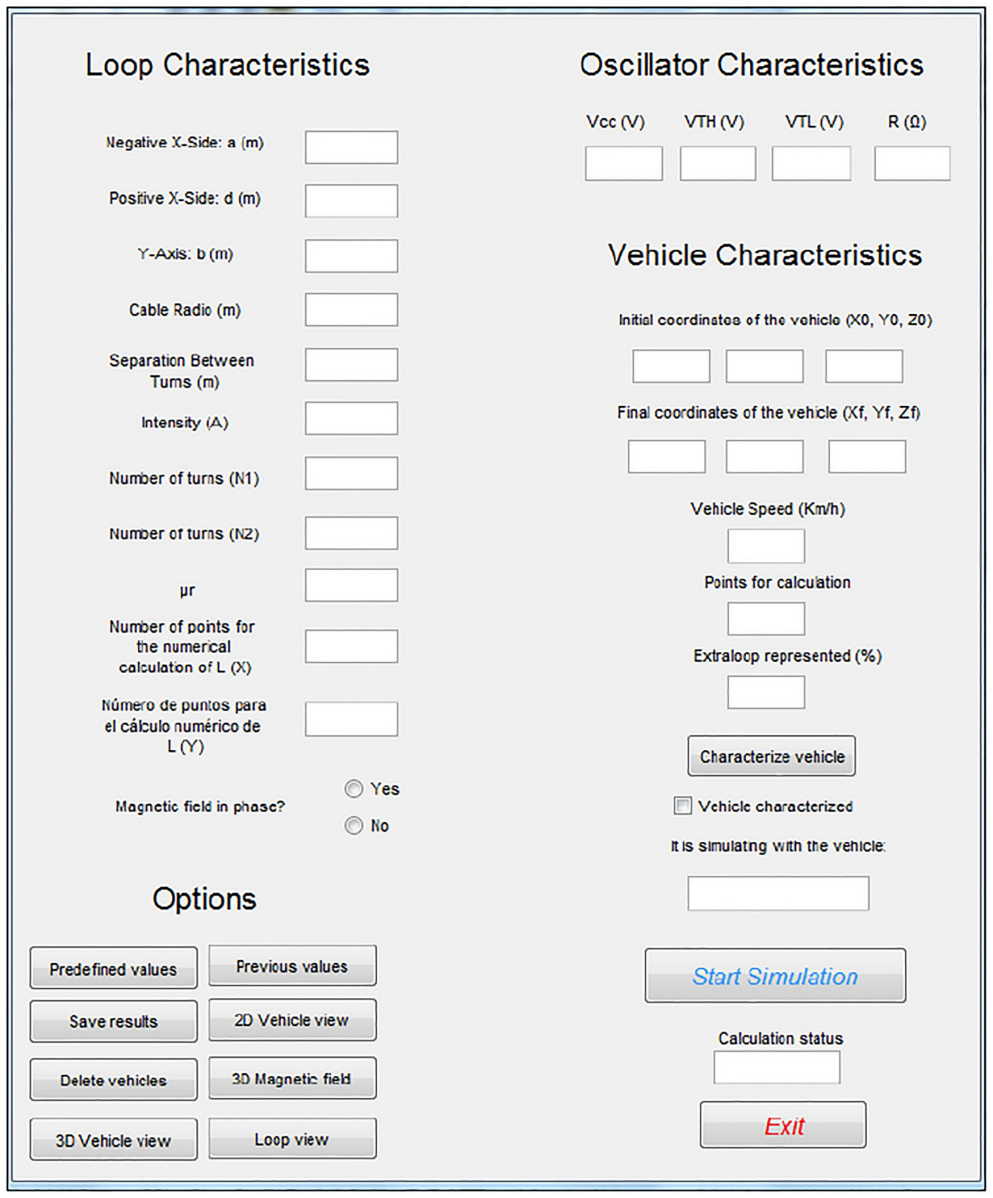
Program that allows the characterization and simulation of vehicles passing over any type of loop to obtain their magnetic profiles.

Notably, with the aim of simplifying the calculation, it can be considered that the vehicle moves centered with respect to the Y-axis. However, this is mostly true in real environments. The appearance of the program developed by our research group is shown in Fig 13.

To compare the magnetic profiles, we worked with real and simulated rectangular loops of dimensions 2 × 2 meters; 3 turns were built with copper wire with a radius of 0.75 mm and a separation of 1.9 mm between turns. The oscillator circuit, whose model can be seen in our previous paper [36], had a resistance of 15 *Ω*, a supply voltage of 5 *V*, a drop of base-emitter voltage of 0.6 *V* and switching voltages of 1.8 *V* and 0.95 *V*.

For the choice of vehicles, we opted for three well-differentiated vehicles whose magnetic profile was registered from previous studies. In this way, we decided to work with a passenger car, a van and a bus:

- Citroën AX (Length = 3.525 *m*. Width = 1.555 *m*. Height = 1.355 *m*).
- Citroën C-15 (Length = 3.995 *m*. Width = 1.655 *m*. Height = 1.800 *m*).
- Bus (Length = 12.080 *m*. Width = 2.500 *m*. Height = 3.120 *m*).

For all cases, the following will be shown:

- A schematic image of the vehicle.
- The parameters used for the simulation.
- The inductance values of the loop located on the road and the vehicle.
- The mutual inductance between the loop located on the road and the vehicle, calculated by the Grover’s methods, as described in the previous section.
- The real magnetic profile (registered by SCT-IL v2.0 equipment developed by our research group whose details are given in [36]), the simulated magnetic profile obtained by considering the vehicle as a single loop rectangular and the simulated magnetic profile obtained by modeling the vehicle as multiple loops of different dimensions.

### Citroën AX

**Fig 14:**
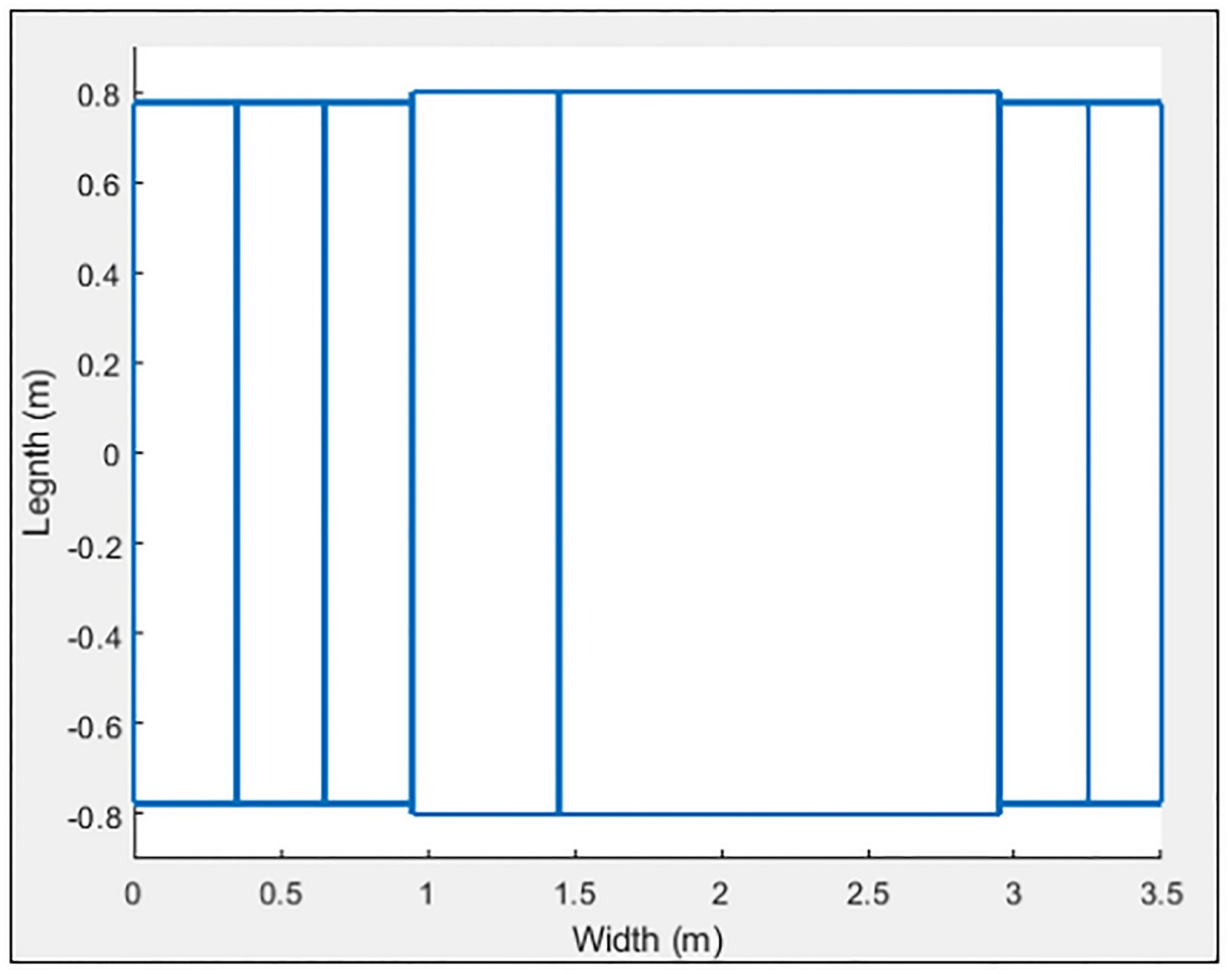
Citroën AX characterized in 7 sections.

**Fig 15:**
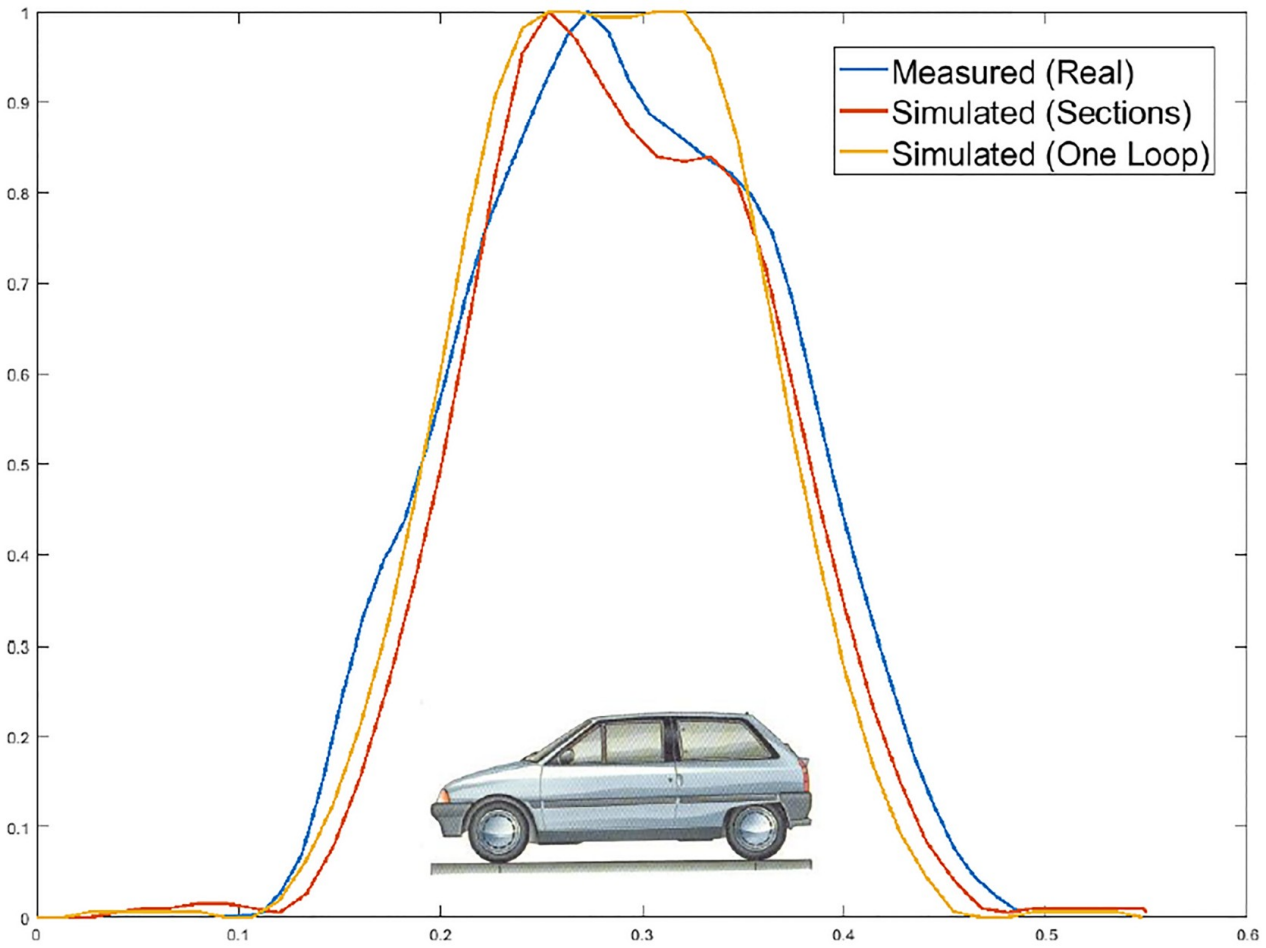
Citroën AX simulation.

The loop and oscillator characteristics are shown in Tables 1 and 2, respectively; the values referring to its dimensions and simulation options are shown in Tables 3 and 4, respectively; the vehicle modeling is shown in Fig 14 and the results are shown in Fig 15 and Table 5.

**Table 1:** Values entered in the program of Fig 13 according to the nomenclature presented in [3,4–3,7].

Loop Characteristics	Value
N_EGATIVE X-SIDE (m)_	1
P_OSITIVE X-SIDE (m)_	1
Y_AXIS (m)_	1
C_ABLE RADIO (m)_	0.00075
S_EPARATION BETWEEN TURNS (m)_	0.0019
I_NTENSITY (A)_	0.1
N_UMBER OF TURNS_ (*N*_1_)	3
N_UMBER OF TURNS_ (*N*_2_)	0
*μ*_*r*_	1
N_UMBER OF POINTS FOR THE NUMERICAL CALCULATION OF L(X)_	889
N_UMBER OF POINTS FOR THE NUMERICAL CALCULATION OF L(Y)_	889
M_AGNETIC FIELD IN PHASE_	Yes

**Table 2:** Oscillator values.

Oscillator Characteristics	Value
V_CC(V)_	4.4
V_TH(V)_	1.8
V_TL(V)_	0.95
R_(Ω)_	15

**Table 3:** Citroën AX simulation values.

Simulation Characteristics	Value
X_0_	2.95
Y_0_	0
Z_0_	0.5
X_F_	-2.95
Y_F_	0
Z_F_	0.5
V_EHICLE SPEED (km/h)_	50
C_ALCULATION POINTS_	50
E_XTRA LOOP REPRESENTED (%)_	0

**Table 4:** Vehicle modeling—Citroën AX.

Section	Length (m)	Width (m)	Height (m)
S_1_	0.35	1.55	0.375
S_2_	0.3	1.55	0.385
S_3_	0.3	1.55	0.39
S_4_	0.5	1.6	0.45
S_5_	1.5	1.6	0.475
S_6_	0.3	1.55	0.45
S_7_	0.25	1.55	0.48

**Table 5:** Results for Citroën AX.

Section	Value (μH)
L_LOOP_	92.05
L_VEHICLE_	14.71

### Citroen C-15

**Fig 16:**
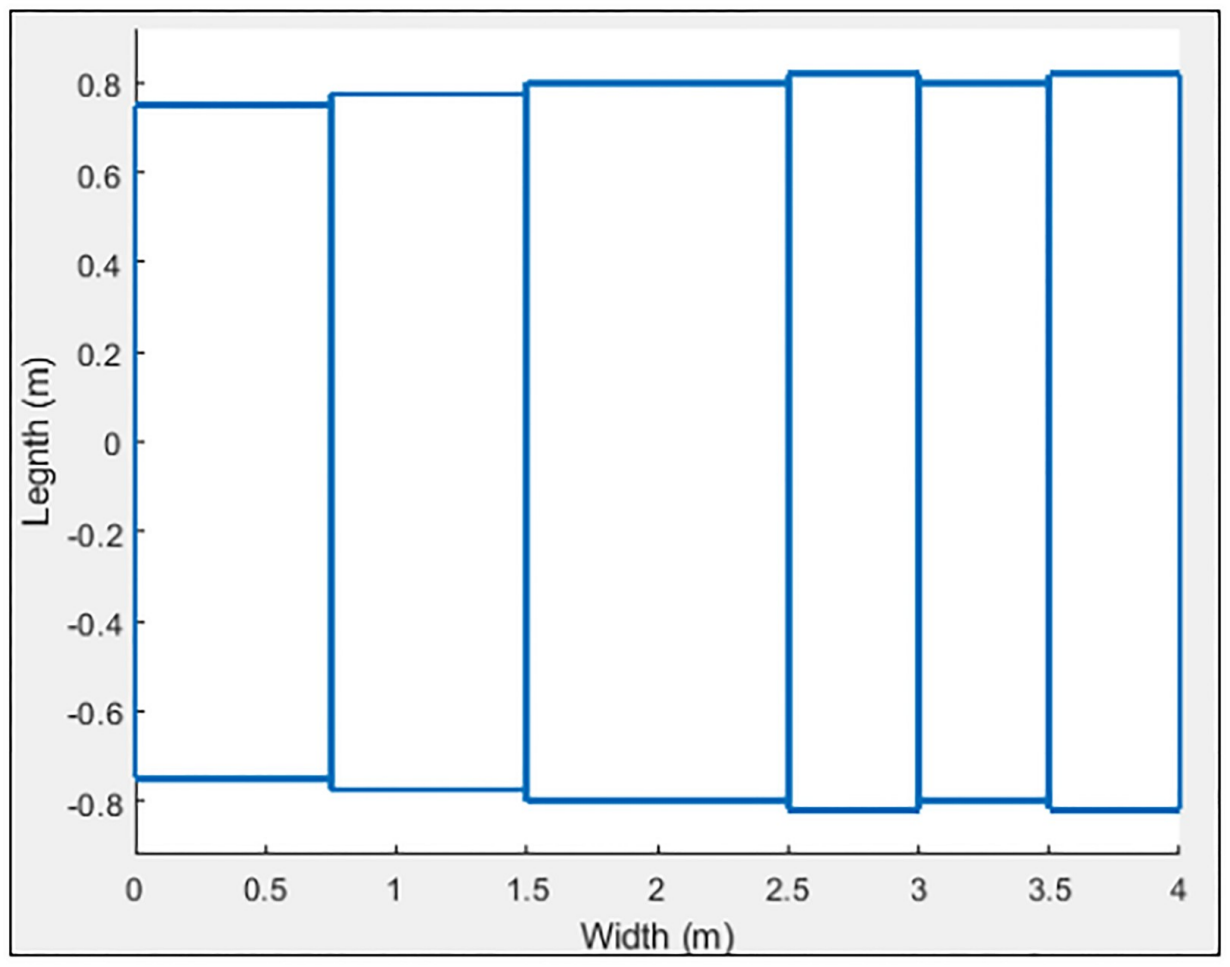
Citroën-15 characterized in 6 sections.

**Fig 17:**
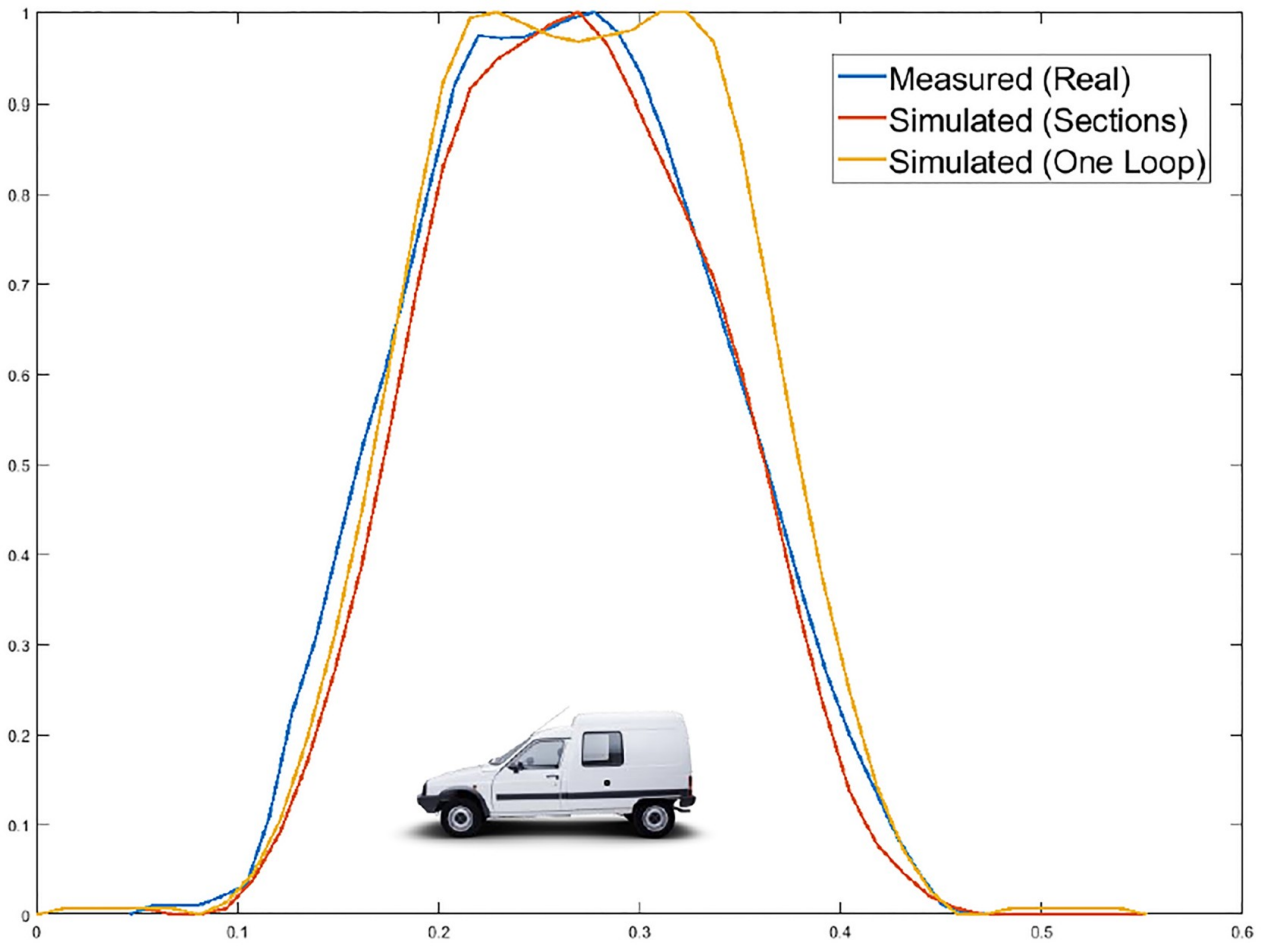
Citroën C-15 simulation.

To carry out the analysis of this van, the loop and oscillator characteristics were the same as those in Tables 1 and 2 respectively. The only values that changed were those referring to its dimensions and simulation options, which are shown in Tables 6 and 7, respectively. The vehicle modeling is shown in Fig 16, and the results are shown in Fig 17 and Table 8.

**Table 6:** Citroën C-15 simulation values.

Simulation Characteristics	Value
X_0_	3.2
Y_0_	0
Z_0_	0.5
X_F_	-3.2
Y_F_	0
Z_F_	0.5
V_EHICLE SPEED (km/h)_	50
C_ALCULATION POINTS_	50
E_XTRA LOOP REPRESENTED (%)_	0

**Table 7:** Vehicle modeling—Citroën C-15.

Section	Length (m)	Width (m)	Height (m)
S_1_	0.75	1.5	0.4
S_2_	0.75	1.55	0.3975
S_3_	1	1.6	0.395
S_4_	0.5	1.64	0.3925
S_5_	0.5	1.6	0.65
S_6_	0.5	1.64	0.6

**Table 8:** Results for Citroën C-15.

Section	Value (μH)
L_LOOP_	92.05
L_VEHICLE_	16.71

### Bus

**Fig 18:**
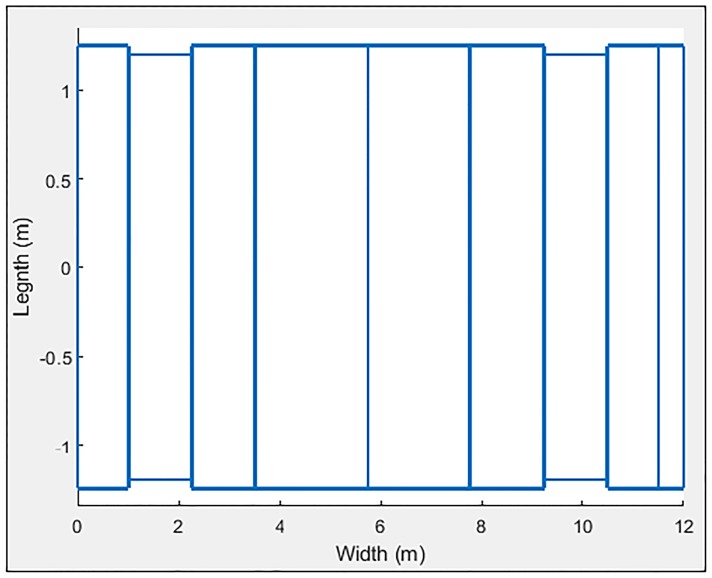
Bus characterized in 9 sections.

**Fig 19:**
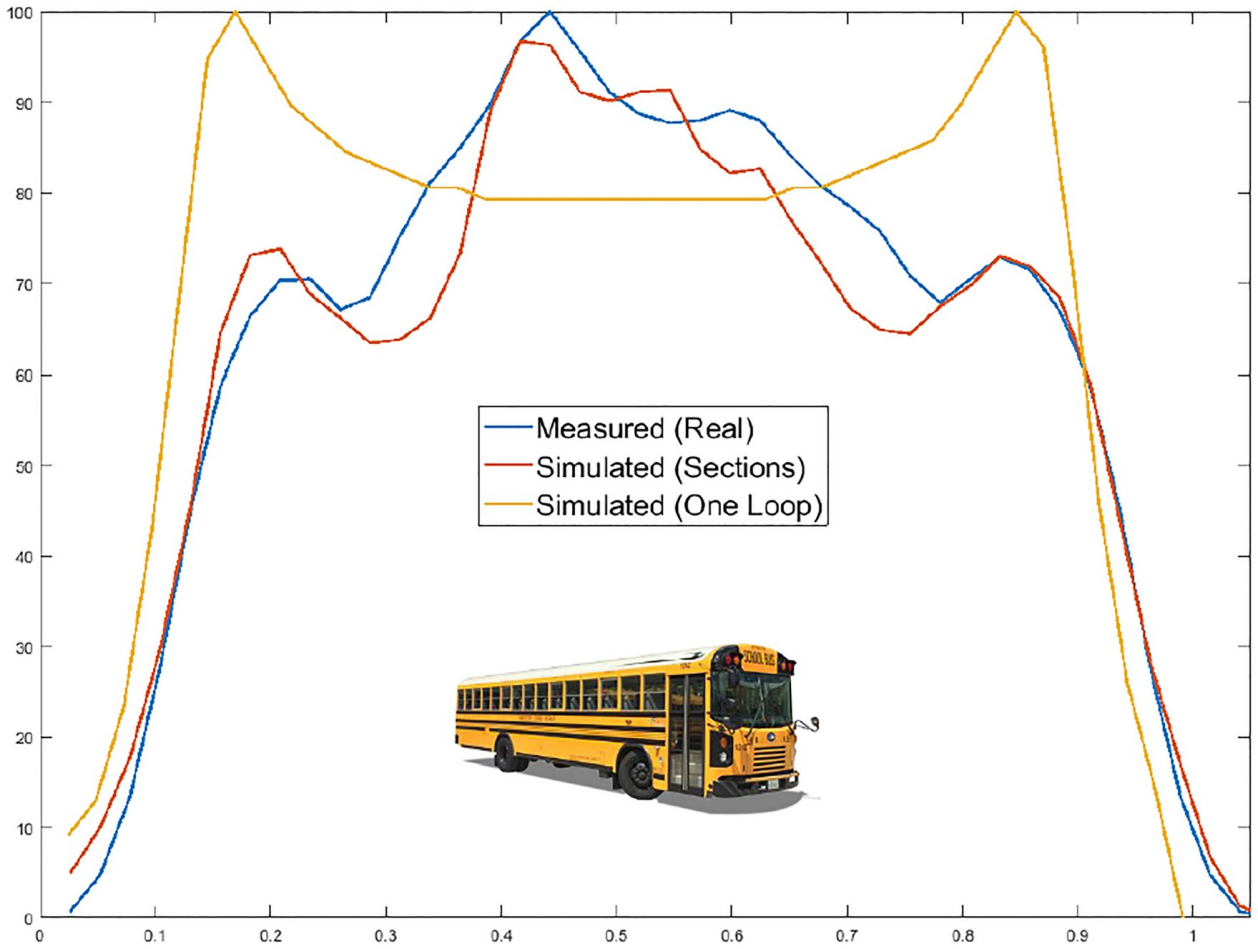
Bus simulation.

To carry out the analysis of this bus, the loop and oscillator characteristics were the same as those in Tables 1 and 2, respectively. The only values that changed were those referring to their dimensions and simulation options, which are shown in Tables 9 and 10, respectively. The vehicle modeling is shown in Fig 18, and the results are shown in Fig 19 and Table 11.

**Table 9:** Bus simulation values.

Simulation Characteristics	Value
X_0_	6.9
Y_0_	0
Z_0_	0.5
X_F_	-6.9
Y_F_	0
Z_F_	0.5
V_EHICLE SPEED (km/h)_	50
C_ALCULATION POINTS_	50
E_XTRA LOOP REPRESENTED (%)_	0

**Table 10:** Vehicle modeling—Bus.

Section	Length (m)	Width (m)	Height (m)
S_1_	1	2.5	0.8
S_2_	1.25	2.4	0.7
S_3_	1.25	2.5	0.65
S_4_	2.25	2.5	0.525
S_5_	2	2.5	0.55
S_6_	1.5	2.5	0.65
S_7_	1.25	2.4	0.7
S_8_	1	2.5	0.8
S_9_	0.5	2.5	0.7

**Table 11:** Results for Bus.

Section	Value (μH)
L_LOOP_	92.05
L_VEHICLE_	44.83

## Statistical analysis

To verify the usefulness and effectiveness of our modeling, a statistical analysis based on the previous information was carried out. For this purpose, the following data were used:

- The magnetic profile measured by SCT-CEM-4 device (this equipment is an improved version of the SCT-IL v2.0 system developed by the Traffic Control Systems Group of the ITACA Institute of the Polytechnic University of Valencia, which is patented with the application number P200401111 and whose details are given in [36]).
- The simulated magnetic profile when the vehicle is modeled as one single section.
- The simulated magnetic profile when the vehicle is modeled as several sections.

In this way, the maximum deviation as well as the mean and the standard deviation are provided in Table 12. The first column of each vehicle corresponds to the data calculated from its simulation as a simple metal plate, while the second column, which includes an asterisk *, corresponds to the values calculated by our new modeling method.

**Table 12:** Statistical analysis. Vehicles which include * correspond to sectioned vehicles.

	Citroën AX	Citroën AX*	Citroën C-15	Citroën C-15*	Bus	Bus*
M_AXIMUM DEVIATION_	14.5	8.8	34.7	9.8	30.6	14.9
M_EAN_ D_EVIATION_	-0.4	0.6	5.8	2.2	13.4	1.6
S_TANDARD_ D_EVIATION_	6.8	3.2	12.3	3.0	13.3	5.2

## Conclusions

Improving the current infrastructure and user mobility and providing services for smart cities is one of the main priorities in Intelligent Transportation Systems (ITS). For this reason, simulations of different road situations related to these new traffic sensors have emerged as a necessity in present-day society. In fact, computer simulations have become essential for modern engineering. Developing and applying simulation techniques has accelerated the understanding of processes, and therefore, explaining, improving or testing any phenomenon is currently much easier.

Thus, we have designed a new vehicle modeling for the analysis of the response of detectors based on inductive loops, whose results show that simulations are greatly improved when multiple sections are used. Our study has demonstrated something that could already be observed in Fig 2: the greater the dimensions and complexity of the vehicle, the worse the result obtained when simulating with a single section. Consequently, it is clear that in any of the three situations analyzed, modeling by sections always provides a better result, but it is especially evident in large vehicles such as vans, trucks and buses. This can be seen both visually and numerically.

Therefore, from now on, we recommend using this alternative model based on polyhedral vehicle modeling capable of sectioning vehicles as multiple loops of different dimensions instead of the inefficient vehicle modeling used today. For this purpose, we have analyzed the magnetic characteristics of the new modeling and have offered mathematical expressions that allow us to obtain all the necessary inductance values. Moreover, the indicated expressions have been implemented in a computer program developed by the authors that allows the simulation of any situation.

In this way, it has become clear that the proposed vehicle modeling is much closer to the real magnetic profiles than the modeling used to date, and therefore, it will help the development of new features of this reference sensor. This new model could faithfully characterize the magnetic profile of vehicles and thus, be used for different applications such as stolen vehicle location, green wave generation for priority vehicles, cooperative crossing collision prevention system or access control systems [38–41]. However, future work will focus on finding an algorithm that automatically is capable of optimally sectioning any given vehicle.

## Supporting information

S1 File Magnetic profiles—Fig 1. This file contains an Excel in which the time and frequency variation data used to elaborate Fig 1 are shown. These data were recorded by SCT-CEM-4 device, an improved version of the SCT-IL v2.0 system developed by the Traffic Control Systems Group of the ITACA Institute of the Polytechnic University of Valencia, which is patented with the application number P200401111.
(XLSX)

S2 File Statistical analysis. This file contains an Excel where all the data involved in the statistical analysis are shown. In addition, new graphs that show the errors committed in both methods are included.
(XLSX)
